# *Numb*-exon3 and full length *Numb* equivalently alleviate cholestatic liver fibrosis by inhibiting ductular reaction

**DOI:** 10.1038/s41598-025-23696-3

**Published:** 2025-11-14

**Authors:** Yan-nan Xu, Meng-yao Zong, Wen Xu, Shi-hao Zhang, Dan-yang Wang, Xin-rui Zheng, Fei-fei Xing, Jun-yi Zhan, Jia-mei Chen, Gao-feng Chen, Ping Liu, Wei Liu, Yong-ping Mu

**Affiliations:** Institute of Liver Diseases, Key Laboratory of Liver and Kidney Disease of the Ministry of Education, Shuguang Hospital affiliated to Shanghai University of Traditional Chinese Medicine (TCM), Shanghai University of TCM, Clinical key laboratory of TCM of Shanghai, 528, Zhangheng Road, Shanghai, 201203 Pudong district China

**Keywords:** Cholestatic liver fibrosis, Numb, *Numb-*Exon3, Ductular reaction, Liver regeneration, Liver fibrosis, Primary biliary cirrhosis

## Abstract

Cholestasis can occur in various acute and chronic liver diseases, with primary biliary cholangitis (PBC) and primary sclerosing cholangitis (PSC) being the most common clinical manifestations. Without appropriate treatment, these conditions may ultimately progress to liver cirrhosis and hepatic failure. Therefore, identifying novel therapeutic targets is of great importance. Our previous research had found a gene target named *Numb* which is a determinant of stem cell fate can increase the anti-cholestatic liver fibrosis (CLF) effect of bone marrow mesenchymal stem cells (BM-MSCs). However, whether the *Numb* gene or its exon has direct anti-CLF activity is unclear. In this study, an adeno-associated virus (AAV) was used as a gene delivery vector to overexpress the full-length *Numb* gene directly in the rat liver. In addition, Exon3 was overexpressed to clarify the effective site of *Numb* gene for comparison. AAV.*Numb* can alleviate CLF and suppressed the activation of Notch signaling and the differentiation of hepatic progenitor cells (HPCs) into biliary epithelial cells (BECs), and the anti-CLF effect of *Numb*-Exon3 was similar to that of full-length *Numb*. The findings revealed that *Numb* gene may be a new therapeutic target for PBC and that Exon3 may be an effective site.

**Supplementary Information:**

The online version contains supplementary material available at 10.1038/s41598-025-23696-3.

## Introduction

Cholestatic liver disease is caused by genetic defects, mechanical abnormalities, toxins, or immune system dysregulation, which damage the bile ducts, leading to bile accumulation and liver tissue injury^1^. Its primary pathological features include inflammatory destruction of intrahepatic bile ducts and abnormal proliferation of small bile ducts, ultimately resulting in cholestatic liver fibrosis (CLF), cirrhosis, and even liver cancer^2^. Currently, there is a lack of effective clinical treatments for CLF. Although ursodeoxycholic acid (UDCA) can improve the quality of life of patients with primary biliary cholangitis (PBC), it can not halt the progression of liver fibrosis^3^. Additionally, significant obstacles remain in the development of biologics for CLF treatment. By understanding bile acid metabolism, biliary epithelial cell (BEC) biology, immune-mediated bile duct attack, and fibrogenesis, new therapeutic targets can be identified for the development of biotherapies, including gene therapy^4^.

Targeting genes involved in hepatic progenitor cell (HPC) activation and bile duct proliferation during ductular reaction (DR) may be a promising strategy. Among these, the Notch signaling pathway, critical for normal embryonic development and stem cell maintenance, is reactivated in CLF and is associated with its pathogenesis and the differentiation of HPCs^5^. Numb, a key cell fate determinant, acts as a negative regulator of the Notch pathway^6^ and is closely linked to signaling pathways mediating abnormal BEC proliferation, such as Notch, Wnt, and Hedgehog^7^. Some researchers propose that Numb protein may function as a “switch” between the Wnt and Notch pathways, ultimately determining whether HPCs differentiate into BECs or hepatocytes. If these regulatory mechanisms are disrupted, reactive BECs become activated, accompanied by persistent inflammatory responses, stromal cell activation, and portal fibrosis, leading to progressive liver disease^8^.

Our previous studies confirmed that the expression level of Numb was significantly reduced in the livers of rats with CLF and PBC patients. In addition, bone marrow mesenchymal stem cells (BM-MSCs) that overexpress the *Numb* gene differentiate into hepatocytes, thereby inhibiting CLF progression. Conversely, BM-MSCs with *Numb* knockdown differentiated into BECs, thereby promoting the DR and progression of CLF^5,9^, suggesting that the *Numb* gene may become a tool for the treatment of CLF. In mammals, the *Numb* gene produces four Numb protein subtypes (Numb1-4) through alternative splicing of Exon3 and Exon9^10^. Numb proteins can be classified into long phosphotyrosine binding (PTB) subtypes (Numb-PTB^L^, including Numb1/2) and short PTB subtypes (Numb-PTB^S^, including Numb3/4) on the basis of whether the PTB domain contains the Exon3 coding region^11^. To date, the physiological relevance of Numb splicing isoforms and their precise regulatory mechanisms remain poorly understood. Nevertheless, accumulating evidence has revealed distinct biological functions among different Numb variants. For example, in PC12 cells, the Numb isoforms lacking the PTB domain exhibited higher Notch activity compared to those containing the complete PTB domain^11^. These findings indicate that the PTB domain encoded by Exon3 is essential for Numb to exert its anti-Notch activity. In the present study, we first confirmed the anti-CLF effect of the *Numb* gene. Furthermore, we compared the differences in the anti-CLF effects between full-length *Numb* and *Numb*-Exon3. The results revealed that *Numb* determines the fate of HPCs and alleviates the progression of CLF. What’s more, *Numb*-Exon3 has the same effect on the CLF as does full-length *Numb* and may be an effective site.

Gene therapy is the introduction of genetic material into a patient’s body to alter gene or protein expression to provide therapeutic effects, and it has become an increasingly popular field. Several treatments utilizing gene transfer mechanisms have been developed, and adeno-associated virus (AAV) vectors have proven to be effective for in-body gene therapy. The introduction of several novel liver-directed treatments for hemophilia A and B based on AAV vectors has generated a great deal of interest in gene therapy within the hepatology community, with the liver increasingly being viewed as a target for gene therapy^12^. Therefore, it is feasible and safe to deliver *Numb* gene which can determine the differentiation of HPCs to the liver via the AAV vector. In this study, a rat CLF model was established by bile duct ligation (BDL) to investigate the therapeutic effect of over-expression of *Numb* gene and *Numb* Exon3 in adult liver with AAV as a carrier.

## Results

### The *Numb* gene reduces liver inflammation and inhibits hepatic stellate cell (HSC) activation and the progression of liver fibrosis

Although *Numb* was confirmed to determine the fate of BM-MSCs in the livers of rats with CLF in our previous studies^9^, we did not determine whether *Numb* supplementation in the adult liver alters the fate of HPCs and the progression of CLF.

**Fig. 1:**
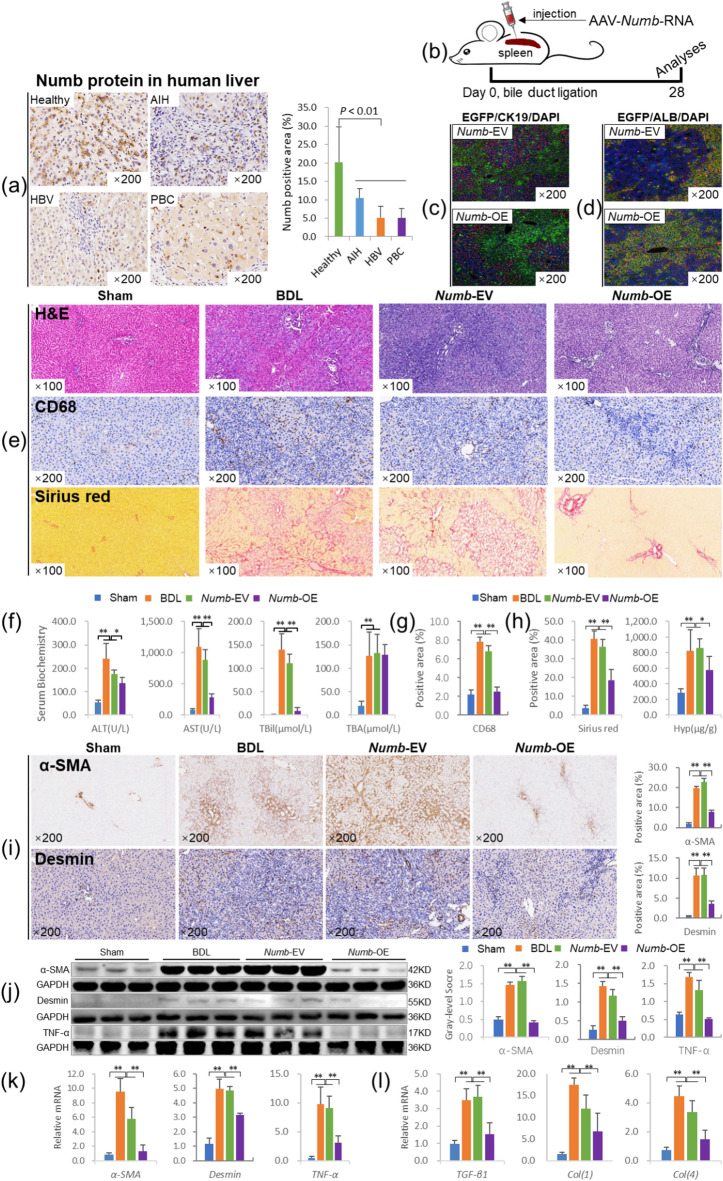
The *Numb* gene inhibits the progression of CLF. (**a**) Expression of Numb in the livers of a healthy population (*n* = 10) and patients with AIH, HBV, and PBC-related cirrhosis (*n* = 20): Numb immunostaining (×200) and its positive area (%). (**b**) Experimental flow chart. (**c**) EGFP (labeled adeno-associated virus)/CK19 coimmunostaining in livers from the CLF group (× 200). (**d**) EGFP/ALB coimmunostaining in livers from the CLF group (× 200). (**e**) H&E staining (× 100), CD68 immunostaining (× 200) and Sirius red collagen staining (× 100). (**f**) Serum levels of biochemical markers. (**g**) positive area (%) of CD68. (**h**) Sirius red-stained area (%) and Hyp content. (**i**) α-SMA and Desmin immunostaining (× 200) and their positive area (%). (**j**) α-SMA, Desmin and TNF-α immunoblotting bands, gray-level integrations. (**k**) mRNA expression levels of *α-SMA*, *Desmin* and *TNF-α* (*n* = 6/per group). (**l**) The mRNA levels of cytokines related to liver fibrosis (*n* = 6/per group). *P < 0.05; **P < 0.01. Sham, sham group; BDL, bile duct ligation group; *Numb*-EV, *Numb*-Empty vector group; *Numb*-OE, *Numb*-overexpression group.

First, we measured Numb protein levels in the livers of patients with autoimmune hepatitis (AIH), hepatitis B virus (HBV), or PBC-related cirrhosis. The result showed that Numb was widely expressed in the livers of healthy people, but its expression was clearly decreased in the livers of patients with AIH, HBV or PBC, as the Numb-positive staining area was reduced by 48%, 75% and 73%, respectively, compared with that in the healthy population (*P* <0.01) (Fig. 1a). These findings indicate that the loss of Numb is closely related to the pathogenesis of liver cirrhosis.

Second, we addressed this question by injecting EGFP-labeled adeno-associated virus serotype 8 (AAV8)-*Numb* clones (*Numb*-overexpression, *Numb*-OE) into the spleen at the same time as BDL and obtained samples at the end of the fourth week (Fig. 1b). Then, we performed EGFP/CK19 and EGFP/ALB coimmunostaining to confirm that AAV8-*Numb* clones were expressed in hepatic parenchyma cells rather than BECs (Fig. 1c and d).

We detected the expression pattern of Numb protein in the liver. Immunofluorescence co-staining of Numb and HEP showed widespread co-localization. The co-localized area was significantly reduced in the BDL and *Numb*-Empty vector (*Numb*-EV) groups compared to the Sham group, while it was markedly increased in the *Numb-*OE group. Numb also exhibited co-localization with CK19, but the fluorescence intensity of Numb on newly formed bile ducts was notably weaker than that on hepatocytes. In terms of co-localized area, it was significantly increased in the BDL and *Numb-*EV groups but markedly decreased in the *Numb-*OE group. The co-localization pattern of Numb/OV6 was consistent with that of CK19 ( Fig. S1-3).

**Fig. 2:**
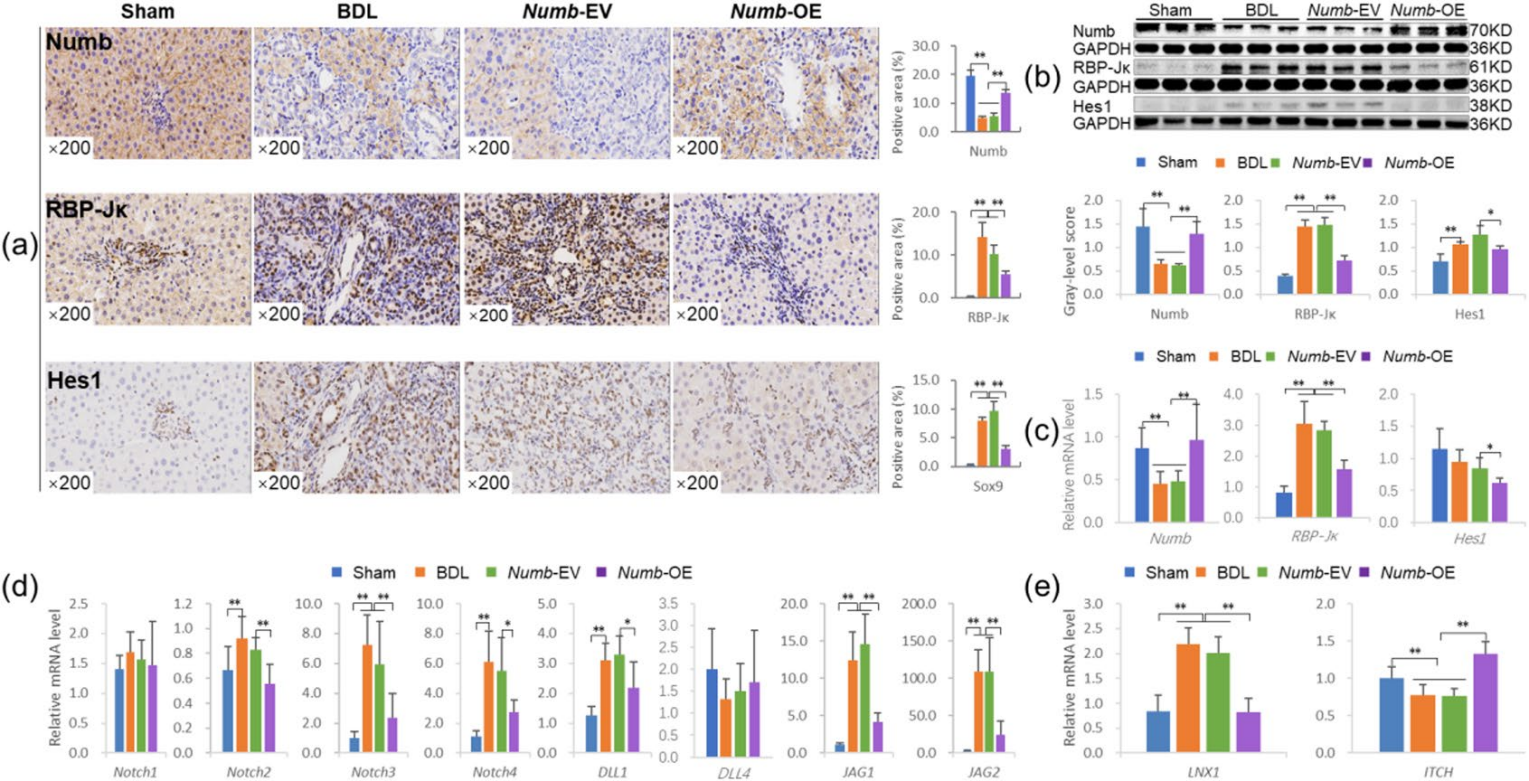
*Numb* suppresses the activation of Notch signaling. (**a**) Numb, RBP-Jκ and Hes1 immunostaining (× 200) and their positive area (%). (**b**) Protein levels of Numb, RBP-Jκ, and Hes1. (**c**) mRNA levels of *Numb*,* RBP-Jκ*, and *Hes1* (*n* = 6/per group). (**d**) mRNA levels of *Notch-1/-2/-3/-4*,* DLL-1/-4*, and *JAG-1/-2* (*n* = 6/per group). (**e**) mRNA levels of *LNX1* and *ITCH* (*n* = 6/per group). *P < 0.05; **P < 0.01. Sham, sham group; BDL, bile duct ligation group; *Numb*-EV, *Numb*-Empty vector group; *Numb*-OE, *Numb*-overexpression group.

**Fig. 3:**
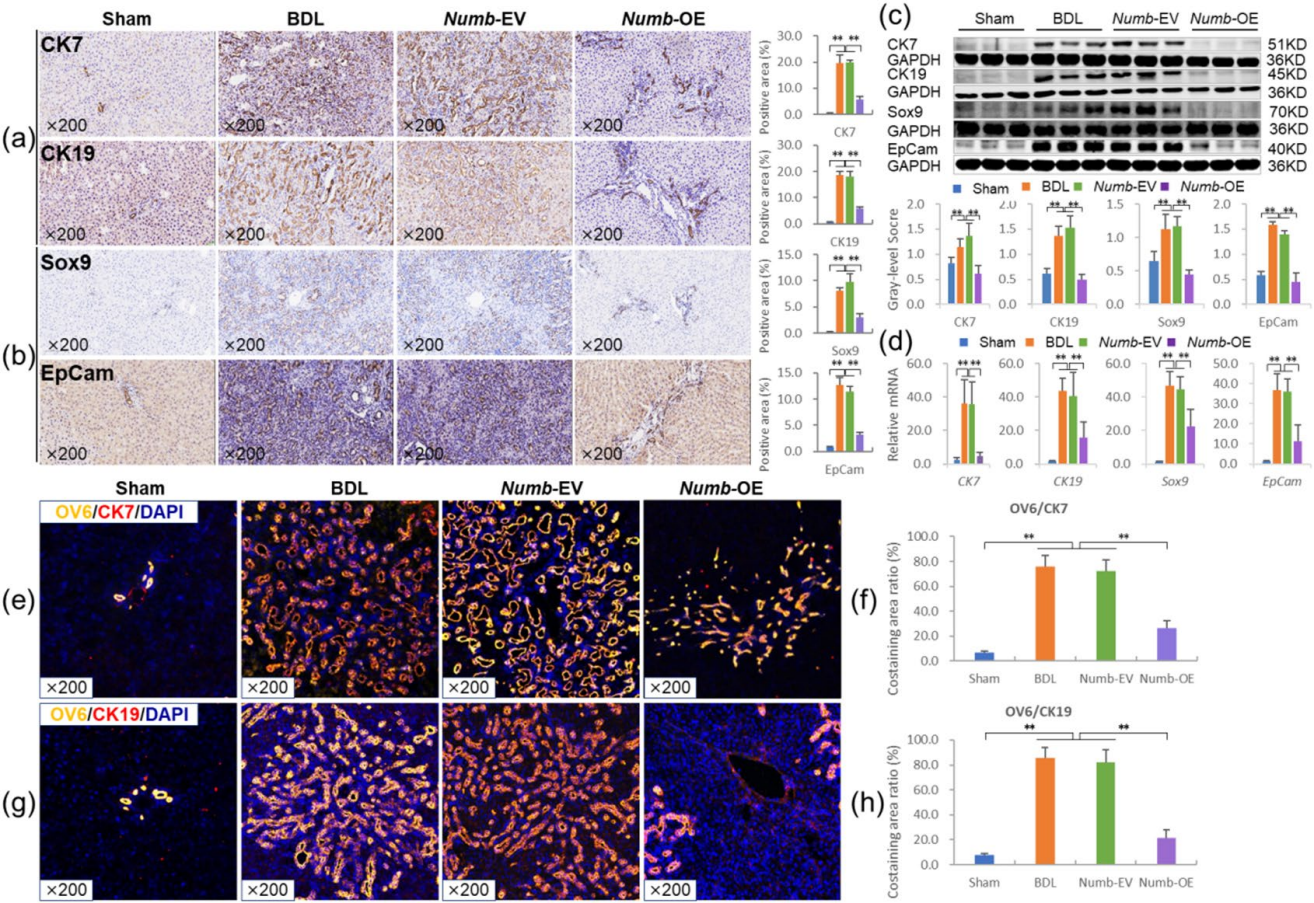
*Numb* inhibits the differentiation of HPCs into BECs. (**a**) CK7 and CK19 immunostaining (× 200) and their positive area (%). (**b**) Sox9 and EpCam immunostaining (× 200) and their positive area (%). (**c**) Protein levels of CK7, CK19, Sox9 and EpCam (*n* = 6/per group). (**d**) mRNA levels of *CK7*,* CK19*,* Sox9* and *EpCam* (*n* = 6/per group). (**e**) OV6/CK7 immunofluorescence costaining (× 200) and (**f**) the costaining area ratio of OV6/CK7. (**g**) OV6/CK19 immunofluorescence costaining (× 200) and (**h**) the costaining area ratio of OV6/CK19. *P < 0.05; **P < 0.01. Sham, sham group; BDL, bile duct ligation group; *Numb*-EV, *Numb*-Empty vector group; *Numb*-OE, *Numb*-overexpression group.

H&E and Sirius red staining showed that the degree of the inflammatory response, bile duct hyperplasia and collagen deposition were clearly reduced in the *Numb*-OE group compared with the *Numb*-EV group (Fig. 1e). Consistent with the histopathological results, the serum ALT, AST and TBil levels were significantly decreased in the *Numb*-OE group compared with the BDL and *Numb*-EV groups (*P* < 0.05 or *P* < 0.01) (Fig. 1f). Moreover, the results of immunostaining for CD68, a marker of pro-inflammatory macrophages, showed that the expression of CD68 in liver tissue was significantly increased in the BDL group and the *Numb*-EV group, while it was significantly decreased in the *Numb*-OE group (Fig. 1e). And the quantitative analysis of positive areas showed consistent trends with the immunostaining results (Fig. 1g). In addition, the area positive for Sirius red staining and Hyp content were significantly decreased in the *Numb*-OE group compared with the BDL and *Numb*-EV groups (*P* < 0.05 or *P* < 0.01) (Fig. 1h).

In addition, immunostaining showed that the expression of α-SMA and Desmin (activated HSC markers^13^ were substantially lower in the livers of the *Numb*-OE group than in those of the *Numb*-EV group, and the quantitative analysis of positive areas showed consistent trends with the immunostaining results (Fig. 1i). Consistent with these results, immunoblotting showed that the protein and mRNA levels of α-SMA, Desmin and tumor necrosis factor alpha (TNF-α) (a proinflammatory cytokine^14^ in the liver were significantly decreased in the *Numb*-OE group compared with the BDL and *Numb*-EV groups (*P* < 0.01) (Fig. 1j, k). Moreover, the mRNA levels of liver fibrosis-related factors, including transforming growth factor beta 1 (*TGF-β1*), collagen I *(Col(1))*, and *Col(4)*, were reduced significantly in the *Numb*-OE group compared with the BDL and *Numb*-EV groups (*P* < 0.01) (Fig. 1l). These results suggest that *Numb* gene supplementation in the adult liver has a good intervention effect on CLF and has the potential to become an effective method for gene therapy of CLF.

### The *Numb* gene suppresses the activation of Notch signaling

Immunostaining showed that Numb expression was markedly increased and that the expression levels of RBP-Jκ (a Notch signaling transcription factor^15^ and Hes1 (a Notch signaling target gene^15^ were markedly lower in the livers of the *Numb*-OE group than in those of the BDL and *Numb*-EV groups, and their quantitative analysis of positive areas showed consistent trends with the immunostaining results (Fig. 2a).

Consistent with the immunostaining results, immunoblotting showed that Numb protein expression was significantly increased in the *Numb*-OE group, whereas the RBP-Jκ and Hes1 protein levels were decreased significantly in the *Numb*-OE group compared with the BDL and *Numb*-EV groups (*P* < 0.01) (Fig. 2b). The trends in the mRNA levels of the *Numb*, *RBP-Jκ* and *Hes1* genes were consistent with the immunoblotting results (Fig. 2c). We also evaluated the mRNA levels of other molecules in the Notch signaling pathway. The mRNA levels of *Notch-2/-3/-4*, *DLL-1* and *JAG-1/-2* were reduced significantly in the *Numb*-OE group compared with the *Numb*-EV group (*P* < 0.05 or *P* < 0.01) (Fig. 2d). In addition, Ligase Numb protein X1 (*LNX1*) mRNA expression was reduced, and E3 ubiquitin-protein ligase Itchy homolog (*ITCH*) mRNA expression was increased significantly in the *Numb*-OE group compared with the *Numb*-EV group (*P* < 0.01) (Fig. 2e). According to these results, *Numb* supplementation in the adult liver may suppress the activation of Notch signaling and the progression of CLF.

### The *Numb* gene inhibits the differentiation of HPCs into BECs

We first determined the expression levels of the DR marker CK7^16^ and the BEC marker CK19^17^. Immunostaining and the quantitative analysis of positive areas showed that the expression of CK7 and CK19 was substantially lower in the livers of the *Numb*-OE group than in those of the BDL and *Numb*-EV groups (Fig. 3a). Consistent with the immunostaining results, the protein and mRNA levels of *CK7* and *CK19* were significantly decreased in the *Numb*-OE group compared with the *Numb*-EV group (*P* < 0.01), suggesting that *Numb* supplementation in the adult liver suppresses the DR (Fig. 3c and d).

We further analyzed the characteristics of BECs and the expression of hepatic stem cell markers to determine the effects of hepatic *Numb* supplementation on HPC differentiation in the livers of rats with CLF. OV6 is an antigen that is specific for rodent HPCs^18^. Sox9 is an endodermal transcription factor, and EpCam is a stem/progenitor cell surface marker^19–21^. Immunostaining showed that the expression of Sox9 and EpCam was markedly lower in the livers of the *Numb*-OE group than in those of the BDL and *Numb*-EV groups and their quantitative analysis of positive areas showed consistent trends with the immunostaining results (Fig. 3b). Consistent with the immunostaining results, the protein and mRNA levels of *Sox9* and *EpCam* were significantly decreased in the *Numb*-OE group compared with the BDL and *Numb*-EV groups (*P* < 0.01) (Fig. 3c and d). Next, the immunofluorescence co-staining results of Ki67 (a proliferation marker) and Sox9 showed significant co-localization in the BDL and *Numb*-EV groups, whereas the co-localized area was markedly reduced in the *Numb*-OE group (Fig. S4). These results suggest that *Numb* supplementation in the adult liver may suppress the activation of HPCs.

To further clarify the effect of *Numb* supplementation in the adult liver on the differentiation of HPCs into BECs, we assessed the coexpression of OV6 and CK7 or OV6 and CK19. Immunofluorescence costaining revealed strong coexpression of OV6/CK7 or OV6/CK19 in proliferating BECs in the BDL and *Numb*-EV groups, and this pathological change was clearly reduced in the *Numb*-OE group (Fig. 3e and g). In addition, we analyzed the positive area ratios of OV6/CK7 and OV6/CK19 immunofluorescence. The results showed that the costaining area ratio of OV6/CK7 in the *Numb*-OE group was 36.3% that in the *Numb*-EV group (26.17% vs. 72.13%, *P* = 0.002) (Fig. 3f), and the costaining area ratio of OV6/CK19 in the *Numb*-OE group was 26.3% that in the *Numb*-EV group (21.58% vs. 82.11%, *P* = 0.001) (Fig. 3h). These results clearly indicate that hepatic *Numb* supplementation inhibits HPCs differentiation into BECs in the livers of rats with CLF and attenuates DR.

These results suggest that *Numb* supplementation in the adult liver effectively inhibits the proliferation of HPCs and their differentiation into BECs, thereby inhibiting the BDL-induced progression of CLF.

In addition, to clarify the impact of *Numb* supplementation on liver regeneration in the adult liver, we measured the expression levels of the hepatocyte markers ALB and HNF4α. The results showed that liver supplementation with the *Numb* gene significantly increased the levels of ALB and HNF4α, as confirmed by serum biochemistry, immunostaining, immunoblotting and qPCR (Fig. S5), suggesting that *Numb* gene supplementation promotes liver regeneration in rats with BDL-induced CLF.

### The *Numb*-Exon3 inhibits HSCs activation and the progression of liver fibrosis

The above studies show that upregulation of *Numb* inhibits the differentiation of HPCs into cholangiocytes, promotes liver regeneration, and promotes the repair of CLF, suggesting that *Numb* gene expression in HPCs is closely related to the occurrence and repair of CLF. The mammalian Numb gene mainly has four isoforms, namely, Numb1-4. Numb proteins can be divided into long PTB subtypes (Numb-PTB^L^, including Numb1/2) and short PTB subtypes (Numb-PTB^S^, including Numb3/4) according to whether the Exon3 coding region is contained in the PTB domain^22^. Some studies have shown that the key exon of the *Numb* gene may be *Numb*-Exon3. However, whether this exon also plays a key role in the occurrence and repair of CLF remains unclear.

**Fig. 4:**
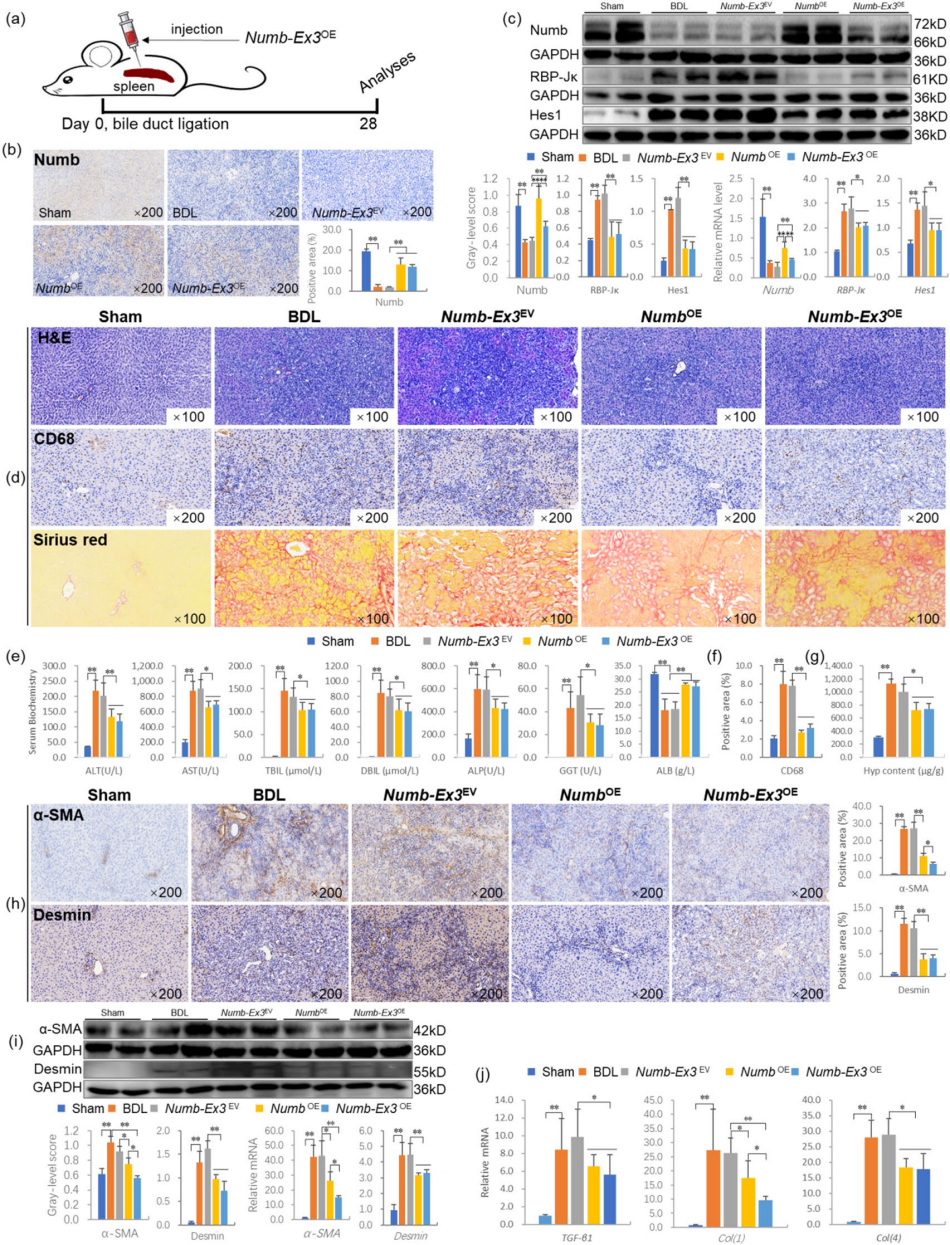
*Numb*-Exon3 inhibits the progression of CLF. (**a**) Experimental flow chart. (**b**) Numb immunostaining (× 200) and its positive area (%). (**c**) Numb, RBP‑Jκ, and Hes1 immunoblotting bands, gray‑level integrations and mRNA expression levels (*n* = 6/per group). (**d**) H&E staining (× 100), CD68 immunostaining and Sirius red collagen staining (× 100). (**e**) Serum levels of biochemical markers. (**f**) positive area (%) of CD68. (**g**) Hyp content in liver tissue. (**h**) α-SMA and Desmin immunostaining (× 200) and their positive area (%). (**i**) α-SMA, Desmin immunoblotting bands, gray-level integrations and mRNA expression (*n* = 6/per group). (**j**) The mRNA levels of cytokines related to liver fibrosis (*n* = 6/per group). *P < 0.05; **P < 0.01. Sham, sham group; BDL, bile duct ligation group; *Numb*-Ex3^EV^, *Numb*-Exon3^Empty vector^ group; *Numb*^OE^, *Numb*^overexpression^ group; *Numb*-Ex3^OE^, *Numb*-Exon3^overexpression^ group.

First, we addressed this question by injecting EGFP-labeled adeno-associated virus serotype 8 (AAV8)-*Numb*-Exon3 clones (*Numb-*Exon3^overexpression^, *Numb-*Exon3^OE^) into the spleen at the same time as BDL and obtaining samples at the end of the fourth week (Fig. 4a). Immunostaining and the quantitative analysis of positive areas showed that the expression of Numb was substantially greater in the livers of the *Numb*-Exon3^OE^ group than in those of the *Numb*-Exon3^Empty vector^ (*Numb*-Exon3^EV^) group. Compared with that in the *Numb*^OE^ group, the expression of Numb was lower in the *Numb*-Exon3^OE^ group (Fig. 4b). Consistent with the immunostaining results, immunoblotting showed that Numb protein expression was increased significantly in the *Numb*-Exon3^OE^ group compared with the *Numb*-Exon3^EV^ group (*P* < 0.01), whereas RBP-Jκ and Hes1 protein levels were decreased significantly in the *Numb*-Exon3^OE^ group compared with the *Numb*-Exon3^EV^ group (*P* < 0.01) (Fig. 4c). Although Numb expression was significantly lower in the *Numb*-Exon3^OE^ group than in the *Numb*^OE^ group (*P* < 0.01), the RBP-Jκ and Hes1 protein levels were similar between the *Numb*^OE^ group and the *Numb*-Exon3^OE^ group. The trends in the mRNA levels of the *Numb*, *RBP-Jκ* and *Hes1* genes were consistent with the immunoblotting results (Fig. 4c).

H&E and Sirius red staining showed that the degree of the inflammatory response, bile duct hyperplasia and collagen deposition were clearly lower in the *Numb*-Exon3^OE^ group than in the *Numb*-Exon3^EV^ group (Fig. 4d). Consistent with the histopathological results, the serum ALT, AST, TBil, direct bilirubin (DBIL), ALP and GGT levels were significantly decreased and the serum ALB levels were significantly increased in the *Numb*-Exon3^OE^ group compared with the *Numb*-Exon3^EV^ group (*P* < 0.05 or *P* < 0.01) (Fig. 4e). Similarly, the results of CD68 immunostaining and the quantitative analysis of positive areas showed that compared to the BDL group and the *Numb*-Exon3^EV^ group, CD68 expression was significantly decreased in the *Numb*^OE^ group and the *Numb*-Exon3^OE^ group. (Fig. 4d, f). In addition, the Hyp content was significantly decreased in the *Numb*-Exon3^OE^ group compared with the *Numb*-Exon3^EV^ group (*P* < 0.05) (Fig. 4g). Moreover, the ALT, AST, TBil, DBIL, ALP, GGT, and ALB levels and the Hyp content were similar between the *Numb*^OE^ group and the *Numb*-Exon3^OE^ group, with no significant differences.

In addition, immunostaining revealed that α-SMA and Desmin expression were substantially lower in the livers of the *Numb*-Exon3^OE^ group than in those of the *Numb*-Exon3^EV^ group (Fig. 4h). Consistent with the immunostaining results, the protein and mRNA levels of α-SMA and Desmin in the liver were significantly decreased in the *Numb*-Exon3^OE^ group compared with the *Numb*-Exon3^EV^ and *Numb*^OE^ groups (*P* < 0.05 or *P* < 0.01) (Fig. i). Moreover, the mRNA levels of liver fibrosis-related factors, including *TGF-β1*, *Col(1)*, and *Col(4)*, were reduced significantly in the *Numb*-Exon3^OE^ group compared with the *Numb*-Exon3^EV^ group (*P* < 0.05 or *P* < 0.01) (Fig. 4j). These results indicate that hepatic *Numb*-Exon3 supplementation inhibits the activation of HSCs.

Therefore, *Numb*-Exon3 supplementation in the adult liver has a good adjuvant effect on CLF, and the effect is similar to that of full-length *Numb*. Thus, *Numb-*Exon3 supplementation may become a new approach for gene therapy for CLF.

### Hepatic *Numb*-Exon3 supplementation inhibits the differentiation of HPCs into BECs

**Fig. 5:**
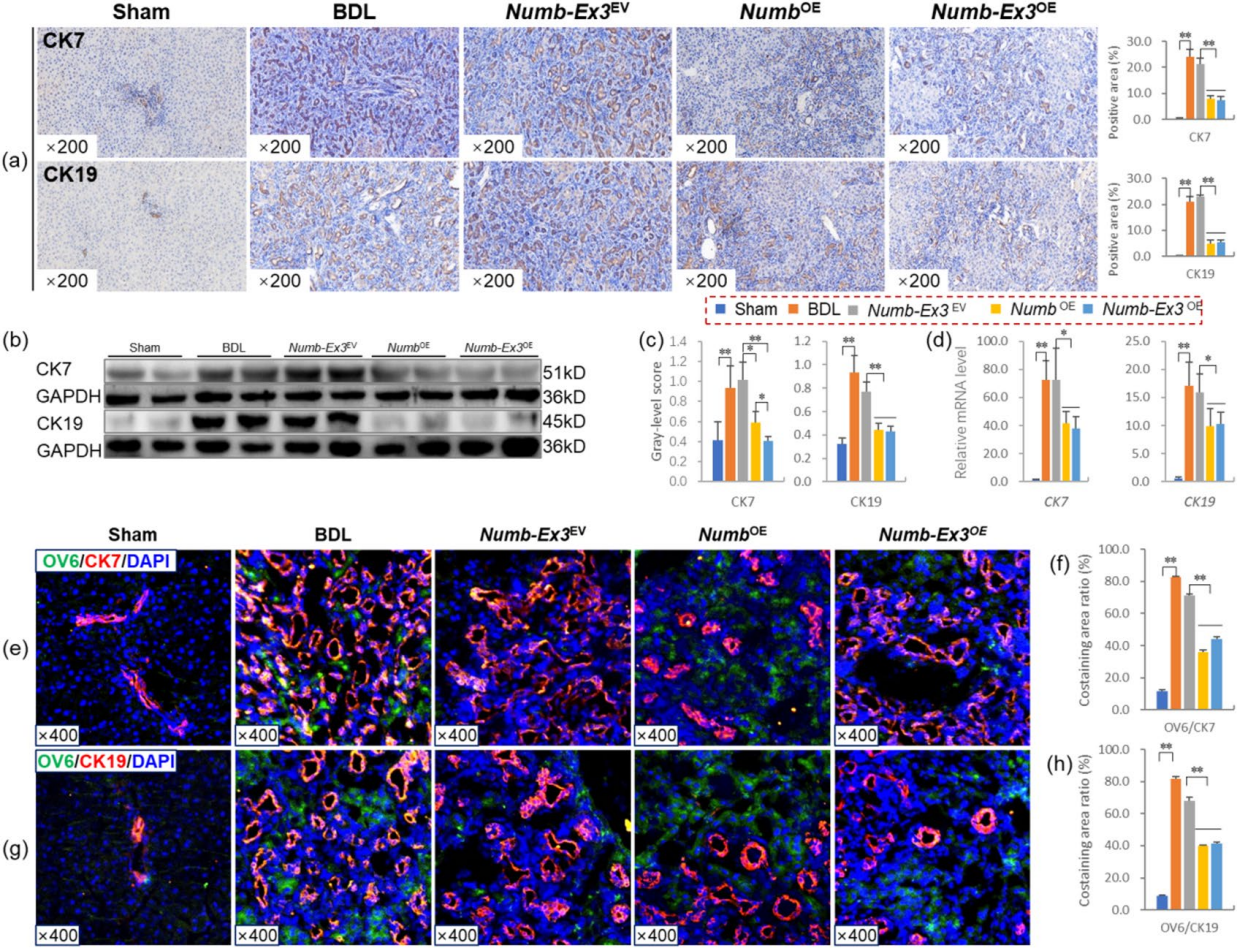
Hepatic *Numb*-Exon3 inhibits the differentiation of HPCs into BECs. (**a**) CK7 and CK19 immunostaining (× 200) and their positive area (%). (**b**) CK7 and CK19 immunoblotting bands and (**c**) gray-level integration. (**d**) mRNA expression levels of *CK7* and *CK19* (*n* = 6/per group). (**e**) OV6/CK7 immunofluorescence costaining (× 200) and (**f**) the costaining area ratio of OV6/CK7. (**g**) OV6/CK19 immunofluorescence costaining (× 200) and (**h**) the costaining area ratio of OV6/CK19. *P < 0.05; **P < 0.01. Sham, sham group; BDL, bile duct ligation group; *Numb*-Ex3^EV^, *Numb*-Exon3^Empty vector^ group; *Numb*^OE^, *Numb*^overexpression^ group; *Numb*-Ex3^OE^, *Numb*-Exon3^overexpression^ group.

Immunostaining and the quantitative analysis of positive areas revealed that the expression levels of CK7 and CK19 were substantially lower in the livers of the *Numb-*Exon3^OE^ group than in those of the *Numb-*Exon3^EV^ group (Fig. 5a). Consistent with the immunostaining results, the protein and mRNA levels of *CK7* and *CK19* were significantly decreased in the *Numb-*Exon3^OE^ group compared with the *Numb-*Exon3^EV^ group (*P* < 0.01). Moreover, the protein and mRNA levels of *CK7* and *CK19* were similar between the *Numb*^OE^ group and the *Numb*-Exon3^OE^ group, suggesting that *Numb-*Exon3 supplementation in the adult liver suppresses the DR, and the effect was similar to that of the full-length *Numb* gene (Fig. 5b-d).

To further clarify the effect of *Numb*-Exon3 on the differentiation of HPCs into BECs, we assessed the coexpression of OV6 and CK7 or OV6 and CK19. Immunofluorescence revealed widespread coexpression of OV6/CK7 or OV6/CK19 in proliferating BECs in the BDL and *Numb*-Exon3^EV^ groups, and these pathological changes were clearly alleviated in the *Numb-*Exon3^OE^ group (Fig. 5e and g). In addition, we determined the positive area ratios of OV6/CK7 and OV6/CK19. The results showed that the costaining area ratio of OV6/CK7 in the *Numb*-Exon3^OE^ group was 38.2% of that in the *Numb-*Exon3^EV^ group (44.04% vs. 71.26%, *P* = 0.000) (Fig. 5f), and the costaining area ratio of OV6/CK19 in the *Numb-*Exon3^OE^ group was 38.9% of that in the *Numb-*Exon3^EV^ group (41.39% vs. 67.79%, *P* = 0.000) (Fig. 5h). Moreover, the costaining area ratios of OV6/CK7 and OV6/CK19 were similar between the *Numb*^OE^ group and the *Numb*-Exon3^OE^ group. These results clearly indicate that hepatic *Numb*-Exon3 supplementation inhibits the differentiation of HPCs into BECs in the livers of rats with CLF and attenuates the DR, and the effect is similar to that of the full-length *Numb* gene. Additionally, immunofluorescence co-staining of Ki67 and Sox9 revealed significant co-localization in the BDL and *Numb-*Exon3^EV^ groups, whereas the co-localized area was markedly reduced in the *Numb*^OE^ group and *Numb*-Exon3^OE^ group (Fig. S4). These results suggest that *Numb*-Exon3 supplementation effectively inhibits the proliferation of HPCs and their differentiation into BECs, thereby inhibiting the progression of BDL-induced CLF.

### *Numb*-Exon3 levels determine the fate of HPCs in vitro

**Fig. 6:**
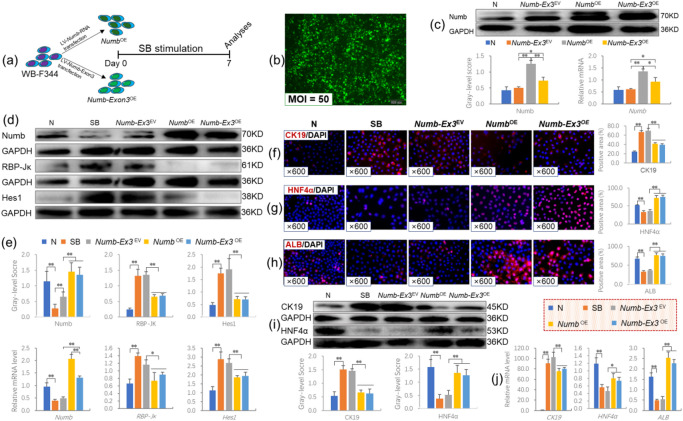
Effects of *Numb*-Exon3 on the differentiation of WB-F344 cells. (**a**) Experimental flow chart. (**b**) Cell morphology and EGFP expression after AAV transfection for 72 h (× 100). (**c**) Verification of the successful overexpression of *Numb* and *Numb-*Exon3 through analysis of Numb protein and mRNA expression levels. (**d**) Numb, RBP‑Jκ, and Hes1 immunoblotting bands and (**e**) gray‑level integrations and mRNA expression levels (*n* = 3/per group). (**f**) CK19 immunofluorescence staining (× 600) and positive area (%). (**g**) HNF4α immunofluorescence staining (× 600) and positive area (%). (**h**) ALB immunofluorescence staining (× 600) and positive area (%). (**i**) CK19 and HNF4α immunoblotting bands and gray‑level integrations (*n* = 3/per group). (**j**) mRNA expression levels of *CK19*, *HNF4α* and *ALB* (*n* = 3/per group). *P < 0.05; **P < 0.01. N, normal group; SB, sodium butyrate group; *Numb*-Ex3^EV^, *Numb*-Exon3^Empty vector^ group; *Numb*^OE^, *Numb*^overexpression^ group; *Numb*-Ex3^OE^, *Numb*-Exon3^overexpression^ group.

To further clarify the regulatory effects of *Numb* or *Numb*-Exon3 on the differentiation of HPCs, we overexpressed *Numb*-Exon3 (*Numb*-Exon3^OE^) or *Numb* (*Numb*^OE^) in WB-F344 cells which have morphological and functional characteristics similar to those of freshly isolated hepatic progenitor cells^23^ and treated them with sodium butyrate to construct an in vitro model that simulates the differentiation of HPCs into cholangiocytes (Fig. 6a).

When the AAV was added at a multiplicity of infection (MOI) of 50, the transfection rate was greater than 80%, and the cell morphology was normal (Fig. 6b). In addition, Numb protein expression was increased significantly in the *Numb*^OE^ and *Numb*-Exon3^OE^ groups compared with the *Numb*-Exon3^EV^ group (*P* < 0.05 or *P* < 0.01), and the *Numb* mRNA level was consistent with the corresponding protein level (Fig. 6c).

Immunoblotting showed that Numb protein expression was significantly increased, whereas RBP-Jκ and Hes1 protein levels were significantly decreased in the *Numb*^OE^ and *Numb*-Exon3^OE^ groups compared with the *Numb*-Exon3^EV^ group (*P* < 0.01). The mRNA levels of *Numb*, *RBP-Jκ* and *Hes1* were consistent with the immunoblotting results (Fig. 6d, e), suggesting that the efficacy of *Numb* and *Numb*-Exon3 in inhibiting the activation of the Notch signaling pathway is equal.

In addition, immunostaining showed that CK19 expression was significantly increased in the SB and *Numb*-Exon3^EV^ groups but significantly decreased in the *Numb*^OE^ and *Numb*-Exon3^OE^ groups (*P* < 0.01), and there was no significant difference between the *Numb*^OE^ and *Numb*-Exon3^OE^ groups (Fig. 6f). Immunofluorescence staining and positive area analysis showed that the expression of HNF4α and ALB decreased significantly in the SB and *Numb*-Exon3^EV^ groups but increased significantly in the *Numb*-Exon3^OE^ and *Numb*^OE^ groups (*P* < 0.01); however, there was no significant difference between the *Numb*-Exon3^OE^ and *Numb*^OE^ groups (Fig. 6g and h). The protein levels of CK19 and HNF4α and the mRNA levels of *CK19*, *HNF4α* and *ALB* were consistent with the immunostaining results (Fig. 6i and j), suggesting that Numb or Numb-Exon3 not only blocks the differentiation of SB-treated WB-F344 cells into the BEC phenotype but also promotes their differentiation into the hepatocyte phenotype.

## Discussion

AAV vector-mediated gene therapy has been proven safe and effective in clinical trials for liver-directed gene treatment. For example, delivering a functional copy of the factor IX gene to hepatocytes has reduced bleeding episodes in patients with hemophilia B^24^. Although studies suggest that AAV vector transduction efficiency in hepatocytes decreases in fibrotic livers, as an intact liver structure is considered essential for AAV-mediated gene delivery^25^, our experiments demonstrated that AAV vectors carrying EGFP successfully entered hepatocytes in most cases, providing a fundamental basis for their therapeutic effect.

Chronic cholestasis initiates three pivotal pathological processes: (1) ductular reaction, (2) impaired hepatocyte regeneration coupled with oxidative stress-induced injury, and (3) HSCs activation mediated by damage-associated molecular patterns (DAMPs)^26^. HPCs, a major source of DR cells^27^, originate at the interface between the hepatic tubular system and the biliary tree—the canals of Hering^28^—and exhibit bipotent differentiation capacity toward both hepatocytic and biliary lineages^29^. In CLF, HPCs become activated, proliferate, and differentiate into reactive ductular cells, forming an “HPC-derived DR” that exacerbates fibrosis^30^. In PBC, HPCs display robust Notch signaling activation, driving biliary differentiation^31^. Given that the* Numb* gene negatively regulates the Notch pathway, this study focuses on whether Numb could serve as a direct therapeutic target for CLF.

Numb is a stem cell fate determinant that regulates the differentiation direction of daughter cells through asymmetric distribution during mitosis^32^. We found that Numb protein levels in liver biopsy samples from PBC-related cirrhosis patients were only 25.3% of those in healthy controls (*P* < 0.001). This phenomenon was similarly significant in BDL-induced CLF models. Although we have previously demonstrated that *Numb* overexpression in BMSCs can inhibit Notch signaling pathway activation, promote BMSCs differentiation into hepatocytes, and alleviate liver fibrosis^9^, there remains no direct evidence regarding the potential therapeutic effects of *Numb* gene and *Numb*-Exon3 on CLF. In this study, we employed AAV vectors to directly overexpress both full-length *Numb* and *Numb*-Exon3 in the liver. The results showed that *Numb* overexpression in adult liver normalized histopathology and liver function, reduced inflammatory responses, and inhibited the progression of CLF.

We then further investigated the impact of *Numb* overexpression in adult liver on HPC differentiation. As previously mentioned, the emergence of DR is closely associated with HPC activation and proliferation. Sox9, Epcam, and OV6 can all serve as markers for HPCs^18–21,33^, while CK7 and CK19 are BECs markers^16,17^. Immunohistochemical results demonstrated that *Numb* overexpression significantly reduced the expression of Sox9 and Epcam, with both protein and gene levels showing the same trend, indicating that *Numb* overexpression suppressed HPCs proliferation and expansion. Furthermore, immunofluorescence co-staining revealed that many CK19^+^ or CK7^+^ DR cells co-expressed OV6, suggesting these DR cells primarily originated from HPCs. Following *Numb* overexpression, the number of these co-expressing cells similarly decreased. Both protein and gene expression levels of CK7 and CK19 were also reduced after *Numb* overexpression. These findings collectively suggest that *Numb* overexpression alleviates HPC-derived DR and reduces neocholangiole formation. Although we did not obtain direct evidence of HPCs differentiation into hepatocytes in this model, the expression levels of hepatocyte markers ALB and HNF4α significantly increased after Numb supplementation, implying that Numb may promote liver regeneration and consequently facilitate the repair of liver fibrosis.

The Notch signaling pathway is an evolutionarily conserved intercellular signaling mechanism primarily composed of four receptors (Notch1-4), five ligands [Jagged (JAG) 1/2 and Delta-like (DIL) 1/3/4], along with transcription factors and DNA-binding proteins, which collectively regulate cellular proliferation, differentiation and apoptosis^34^. Our previous studies demonstrated significant Notch pathway activation in bile duct ligation (BDL) model rats, where its inhibition markedly suppressed HPC-derived DR and alleviated liver fibrosis^5^. In the current study, *Numb* overexpression substantially reduced mRNA expression levels of key Notch receptors (*Notch2/3/4*), ligands (*JAG1/2* and *DLL1*), and downstream transcription factors (*RBP-Jκ* and *Hes1*), collectively inhibiting Notch signaling activation. These results were also confirmed in vitro^9^. However, the precise mechanism of Numb-mediated Notch inhibition remains unclear. Existing evidence suggests their biological antagonism primarily depends on ubiquitination^35^, while the E3 ubiquitin ligase LNX targets Numb for ubiquitination and degradation (thereby reducing its inhibitory effect on Notch)^36^, the cytoplasmic E3 ligase ITCH cooperates with Numb to promote Notch ubiquitination on cell membranes, facilitating degradation of the Notch intracellular domain (NICD) and preventing its nuclear translocation and downstream gene activation^19^. Thus, the Numb-Notch ubiquitination balance may play a crucial role in maintaining hepatic homeostasis. Notably, hepatic Numb supplementation not only significantly decreased *LNX1* mRNA but also increased *ITCH* mRNA expression, indicating *Numb* overexpression simultaneously inhibits self-ubiquitination while promoting Notch ubiquitination.

However, not all Numb isoforms exert inhibitory effects on the Notch signaling pathway. Through alternative splicing of Exon3 and Exon9, the mammalian *Numb* gene generates four protein isoforms (Numb1-4). Intriguingly, studies have found that increased expression of Exon9-containing Numb isoforms actually promotes Notch signaling activation^37^. More importantly, the presence or absence of the PTB domain encoded by Exon3 shows a strong correlation with Notch activity. Research demonstrates that while all four Numb isoforms can bind to the E3 ubiquitin ligase LNX, only those containing Exon3-encoded intact PTB domains undergo ubiquitination and degradation. This process critically depends on the interaction between LNX’s PDZ1 domain and the PTB domain^38^. These findings collectively indicate that the Exon3-encoded PTB domain is essential for Numb-mediated Notch signaling inhibition and consequent anti-CLF effects.

To further validate the functional domain of Numb responsible for its therapeutic effects. In this study, we examined the intervention effect of *Numb*-Exon3 overexpression in the livers of rats transduced with the AAV8 vector on BDL-induced CLF and discovered that its improvement in liver function and histopathology was comparable to that of the full-length Numb gene. By contrasting the expression of the complete Numb protein in the livers of the two groups of animals, we found that the complete Numb protein level in the Numb-Exon3-overexpressing group was significantly lower than that in the Numb-overexpressing group. These data indicate that the anti-CLF therapeutic effects of Numb1/2 resulting from *Numb*-Exon3 overexpression are similar to those of the full-length *Numb* gene. In the in vitro experiments, through LV transfection to overexpress Numb-Exon3 in WB-F344 cells, the ability of *Numb*-Exon3 to inhibit WB-F344 differentiation into BECs and promote WB-F344 differentiation into hepatocytes was similar to that of the full-length *Numb* overexpression group. A comparison of the expression of the Numb protein in the cells revealed that the total Numb protein level in the *Numb*-Exon3-overexpressing cells was also significantly lower than that in the Numb-overexpressing cells. These data further demonstrate that Exon3 is crucial for the therapeutic efficacy of the *Numb* gene.

What‘s more, Exon3, as a functional exon of the *Numb* gene, can achieve therapeutic effects comparable to full-length *Numb* while potentially offering a safer profile. Some reports indicate that Numb-PTB^L^ has tumor-suppressive effects in liver cancer, whereas Numb-PTB^S^ may exhibit tumorigenic properties^39^. Furthermore, Exon9 in the full-length *Numb* gene is crucial for translating the complete proline rich region (PRR) domain. Studies have shown that the intact PRR domain can promote the proliferation rather than differentiation of cancer stem cells, leading to poor prognosis^40,41^. Although there have been no reports of Numb overexpression causing tumors so far, simply overexpressing Numb-Exon 3 might help avoid this potential risk.

In summary, the *Numb* gene plays an important role in the occurrence and repair of CLF, and its key mechanism involves regulating Notch signaling and subsequently determining the differentiation of HPCs in the livers of individuals with CLF. Exon3 is the key exon through which Numb functions. This study provides scientific evidence for improving the treatment of CLF via hepatic Numb-Exon3 supplementation. Therefore, a new finding of this study is that the overexpression of *Numb* or *Numb*-Exon3 in the liver with an AAV vector may provide a shortcut for the treatment of CLF. However, it is a pity that Numb-Exon3 knockout in the adult liver was not successful when CRISPR-Cas9 technology was used, and we are currently investigating the reasons behind this.

## Conclusions

*Numb* is an important determinant of cell fate. In CLF, *Numb* determines the fate of HPCs, promotes their differentiation into hepatocytes and inhibits their differentiation into BECs by suppressing Notch signaling, and Exon3 is the key exon through which *Numb* produces a marked effect. In particular, the results of this study clearly indicate that supplementation with *Numb*-Exon3 in the adult liver can achieve the same effect against CLF as supplementation with the full-length *Numb* gene, which will provide a safer strategy for gene therapy for CLF.

## Materials and methods

### Study design

The goal of this study was to confirm that high expression of the *Numb* gene or *Numb*-Exon3 in the adult liver inhibits the differentiation of HPCs into BECs (DR), which may be a new strategy for the gene treatment of CLF. *Numb* or *Numb-*Exon3 was overexpressed in adult rat livers by transfecting AAV to confirm that *Numb* and *Numb*-Exon3 supplementation in adult livers can effectively inhibit the differentiation of HPCs into BECs, thereby inhibiting the DR and CLF progression. This study aims to provide a safer and more effective approach for gene therapy for CLF by comparing the CLF effects of *Numb* and *Numb*-Exon3.

### Materials

The following antibodies were used for immunohistochemistry and immunoblot analysis: mouse monoclonal antibody to alpha smooth muscle actin (α-SMA; Clone 1A4; Sigma-Aldrich, St. Louis, MO, USA); rabbit polyclonal antibodies to cytokeratin 7 (CK7; 15539-1-AP), CK19 (10712-1-AP) and albumin (ALB; 16475-1-AP) (Proteintech Group Inc., Chicago, IL, USA); mouse monoclonal antibodies to Hepatocyte (HEP; GTX73779) (San Antonio, Texas, USA). mouse monoclonal antibody to Numb (60137-1-Ig) (Proteintech Group Inc., Chicago, IL, USA); mouse monoclonal antibody to OV6 (MAB2020, R&D Systems, Minneapolis, MN, USA); rabbit polyclonal antibody to Numb (ab220362, Abcam Cambridge, UK); mouse monoclonal antibody to Ki67 (ab279653, Abcam Cambridge, UK); rabbit polyclonal antibody to Numb (YT5320, ImmunoWay Biotechnology Company, Newark, DE, USA); mouse monoclonal antibodies to hepatocyte nuclear factor 4 alpha (HNF4α; sc-374229) and Sox9 (E-9, sc-166505) (Santa Cruz Biotechnology, Inc., CA, USA); rabbit polyclonal antibody to RBP-Jκ (5313; Cell Signaling Technology, Danvers, MA, USA); rabbit polyclonal antibodies to Hes1 (ab108937) and EpCam (ab216832) (Abcam, Cambridge, UK); rabbit monoclonal antibodies to Desmin (GB15075-50, Servicebio, Wuhan, China); rabbit monoclonal antibodies to CD68 (ab125212, Abcam Cambridge, UK); mouse monoclonal antibody to glyceraldehyde-3-phosphate dehydrogenase antibody (GAPDH, Chemicon International, Billerica, MA, USA), IRDye 800CW-conjugated donkey to mouse IgG (H + L) (LI-COR Bioscience, San Jose, CA, USA) and IRDye 680RD-conjugated donkey to rabbit IgG (H + L) (LI-COR Bioscience, San Jose, CA, USA).

### Supplementation of *Numb* or *Numb*-Exon3 in the livers of rats with CLF

#### Overexpression of the rat *Numb* gene

*Numb* overexpression (*Numb*-OE) was performed by cloning and transfecting the *Numb* gene. The *Numb*-OE construct was packaged with AAV8 and named AAV8.*Numb* (titer: 8.86 × 10^12^ v.g/mL, Shanghai Genechem Co., Ltd., Shanghai, China), and its target sequence is shown in Supplementary Text 1. The *Numb* empty vector (*Numb*-EV) was used as the negative control, and an empty AAV8 vector (titer: 7 × 10^12^ v.g./mL; Shanghai Genechem Co., Ltd.), whose component sequence was pAAV8-CMV bGI-MSC-eGFP, was used. The *Numb*-OE or *Numb*-EV construct was injected into the rat spleen at the same time as BDL (concentration: 1.6 × 10^11^ v.g./rat).

#### Overexpression of rat *Numb*-Exon3

*Numb*-Exon3 overexpression (*Numb-*Exon3-OE) was performed by cloning and transfecting *Numb*-Exon3. The *Numb*-Exon3-OE construct was packaged via AAV8 and named AAV8.*Numb*-Exon3 (titer: 5.41 × 10^13^ v.g/mL, Shanghai Genechem Co., Ltd., Shanghai, China), and its target sequence is shown in Supplementary Text 2. The *Numb*-Exon3 empty vector (*Numb*-Exon3-EV) was used as the negative control, and an empty AAV8 vector (titer: 7 × 10^12^ v.g./mL; Shanghai Genechem Co., Ltd.), whose component sequence was pAAV8-CMV bGI-MSC-eGFP, was used. The *Numb*-Exon3-OE or *Numb*-Exon3-EV construct was injected into the rat spleen at the same time as BDL (concentration: 1.6 × 10^11^ v.g./rat).

#### Overexpression of *Numb* or *Numb*-Exon3 in WB-F344 cells

*Numb* and *Numb*-Exon3 were separately overexpressed in WB-F344 (*Numb*^OE^, *Numb-*Exon3^OE^) by cloning and transfecting the *Numb* gene or *Numb*-Exon3. Lentiviral vectors (LV) which was named GV703 were labeled with enhanced green fluores cent protein (GFP). LV-*Numb*-RNA (titer: 3 × 10^8^ TU/ ml, Shanghai Genechem Co., Ltd., Shanghai, China) and LV-*Numb*-Exon3(titer: 2.5 × 10^9^ TU/ ml, Shanghai Genechem Co., Ltd., Shanghai, China) were separately transfected into WB-F344 at a multiplicity of infection (MOI) = 50 with the addition of both HitransG-P and enhanced infection solution (ENi. S, Shanghai GeneChem Co., Ltd., Shanghai, China). The component sequence of LV-*Numb*-RNA (LV-*Numb*, 20910-4) is CMV enhancer-NUMB-3FLAG-EF1a-ZsGreen1-T2A-puromycin, while the component sequence of LV-*Numb*-Exon3-RNA (LV-*Numb*-Exon3 98910-11) is Ubi-MCS-3FLAG-SV40-EGFP-IRES-puromycin. In addition, their target sequences are shown in Supplementary Text3 and Text4. The control WB-F344 (*Numb* overexpression-empty vector, *Numb*-Exon3^EV^) was transfected with CON522, an empty lentivirus vector (titer: 2 × 109 TU/ml, Shanghai Genechem Co., Ltd.), whose component sequence is CMV enhancer-MCS-3FLAG-EF1a-ZsGreen1-T2A-puromycin. After transfection for 6 h, the medium was replaced with vector-free medium.

### Animals and experimental protocol

Male SD rats (160–180 g) were purchased from Vital River Laboratory Animal Technology Co., Ltd. (Beijing, China). Animals were maintained in an environment with a constant temperature and supplied with laboratory chow and water ad libitum. All animal experimental protocols were performed in accordance with relevant guideline and regulations and were approved by the Animal Research Committee at Shanghai University of Traditional Chinese Medicine (PZSHUTCM190628002 and PZSHUTCM2305050005). The work has been reported in line with the ARRIVE guidelines.

**Trial 1.** The impact of the *Numb* gene on CLF.

BDL was performed as previously described^5^. Briefly, 24 rats were randomly divided into a sham group (*n* = 6) and a model group (*n* = 18). Model rats were anesthetized with pentobarbital sodium, and laparotomy was performed via a sterile technique. The common bile duct and the left and right hepatic ducts were isolated. The left and right hepatic ducts and the hepatic portal and duodenal sites of the common bile duct were ligated, and the abdomen was closed. In the sham rats, identical surgery was performed, except that the bile duct was not ligated. After BDL, the model rats were randomly divided into the BDL group (*n* = 6), *Numb* empty vector group (*Numb*-EV, *n* = 6) and *Numb* overexpression group (*Numb*-OE, *n* = 6), and a single dose of 5.0 × 10^10^ v.g. of the construct was injected into the spleens of the corresponding groups. The sham and BDL rats were administered the same volume of physiological saline. At the end of 4 weeks, all rats were euthanized with pentobarbital sodium at a dose of 60 mg/kg, and blood and hepatic tissue samples were obtained.

**Trial 2.** Comparison of the effects of the *Numb* gene and *Numb*-Exon3 on CLF.

The modeling method was the same as that in trial 1. After BDL, the model rats were randomly divided into the BDL group (*n* = 6), *Numb*-Exon3 empty vector group (*Numb*-Exon3^EV^, *n* = 6), *Numb* overexpression group (*Numb*^OE^, *n* = 6) and *Numb*-Exon3 overexpression group (*Numb*-Exon3^OE^, *n* = 6), and a single dose of 5.0 × 10^10^ v.g. construct was injected into the spleens of the corresponding groups. The sham and BDL rats were administered the same volume of physiological saline. At the end of 4 weeks, all rats were euthanized with pentobarbital sodium at a dose of 60 mg/kg, and blood and hepatic tissue samples were obtained.

### Serum biochemistry

Serum alanine aminotransferase (ALT), aspartate aminotransferase (AST), total bilirubin (TBil), alkaline phosphatase (ALP), gamma-glutamyltransferase (GGT), total bile acid (TBA), and ALB levels were detected in the clinical laboratory center of Shuguang Hospital affiliated to Shanghai University of TCM.

### Hepatic hydroxyproline (Hyp) content

The Hyp content was determined using the method reported by Jamall et al.^42^, with some modifications.

### Histopathological and immunohistochemical analyses

Paraformaldehyde-fixed specimens were cut into 4-µm-thick sections and stained with 0.1% (w/v) Sirius Red or hematoxylin and eosin (H&E). Immunostaining was performed using previously published methods^43^. Briefly, sections were deparaffinized, washed, and preincubated with a blocking solution, followed by an incubation with antibodies against α-SMA (1:200), CK7 (1:100), CK19 (1:100), Sox9 (1:100), EpCam (1:100), Numb (1:50), RBP-Jκ (1:1,000), Hes1 (1:100), HNF4α (1:50), ALB (1:50), CD68 (1:1000), Desmin (1:500). Sections were then incubated with HRP-conjugated secondary antibodies (1:1,000) and washed. The samples were visualized using DAB with hematoxylin counterstaining and imaged with a Leica SCN400 scanner (Leica Microsystems Inc., Concord, ON, Canada).

For immunofluorescence staining, frozen specimens were cut into 7-µm-thick sections and subjected to immunofluorescence staining to detect the coexpression of EGFP (marked AAV) and CK19 (1:500), EGFP and ALB (1:50), OV6 (1:500) and CK7(1:400), OV6 and CK19, Numb (1:50) and OV6, Numb and CK19, Numb and HEP (1:500), Sox9 (1:1000) and Ki67 (1:500). After an incubation with the primary antibodies, the samples were washed with PBST and incubated with Alexa Fluor 488-conjugated goat to mouse IgG (A11001; Invitrogen, Carlsbad, CA, USA) or Alexa Fluor 594-conjugated goat to rabbit IgG (AB6939; Abcam, Cambridge, UK) secondary antibodies. The nucleus was stained with 4’,6-diamidino-2-phenylindole (DAPI; 1:1,000), and images were captured using an FV10i confocal laser scanning microscope (Olympus, Japan).

### In vitro experimental protocol

 In vitro studies were performed in WB-F344 cell lines, which have morphological and functional characteristics similar to those of freshly isolated HPCs^23^. WB-F344 cells were divided into the normal group (N), SB group (3.75 mM, Sigma, B5887-1G)^44^ overexpression-empty vector group (*Numb*-Exon3^EV^, negative control), *Numb* overexpression group (*Numb*^OE^, positive control), and *Numb*-Exon3 overexpression group (*Numb*-Exon3^OE^) (*n* = 3 per group).

Cell culture was performed using our previously reported methods^9^. Briefly, cells were cultured at 37 °C in a 5% CO_2_ in air atmosphere with Ham’s F12 medium (Life Technologies) supplemented with 10% fetal calf serum (Life Technologies). Chemically induced differentiation was induced by culturing WB-F344 cells on six-well Permanox Lab-Tek culture slides (NalgeNunc International, Naperville) at a density of 3 × 10^4^ cells/well, starting 24 h after seeding. When the degree of confluence reached 30%, lentiviral transfection was performed (MOI = 50). AAV-*Numb*-Exon3-RNA and transfection was performed as described above. Then, the culture medium was changed to 10% FBS/DMEM after 6 h of transfection and culture continued until 48 h. SB (3.75 mM) was added to the model group and each intervention group to induce differentiation. The culture medium was exchanged every 2 days, and the cells were collected on the 7th day. The immunofluorescence staining method was the same as described above.

### Real-time PCR (RT–PCR)

The mRNA expression levels of *α-SMA*,* Col(1)*,* Col(4)*, *TNF-α*, *TGF-β1*, *CK7*,* CK19*,* Numb*,* RBP-Jκ*,* Hes1*,* Notch-1/-2-/3/-4*,* JAG-1/-2*,* DLL-1/-4*,* JAG-1/-2*,* Sox9*,* EpCam*,* LNX1*,* ITCH*, *HNF4α*,* ALB* and *Desmin* were assessed using RT–PCR. Total RNA was extracted from frozen hepatic tissues using Isogen (TOYOBO, Kita-ku, Osaka, Japan), and RNA from each sample was reverse transcribed using SuperScript II Reverse Transcriptase (Thermo Fisher Scientific, Waltham, MA, USA). The samples were then analyzed using fluorescence-based RT–PCR and SYBR Green Real-Time PCR Master Mix (TOYOBO) according to the manufacturer’s protocols. Primers and oligonucleotide probes were designed using Primer Express (Takara Chemical) and are listed in Table 1. Each PCR amplification was performed on samples from six rats in both the experimental and control groups, or WB-F344 cells cultured in vitro. Individual gene expression was normalized to GAPDH. The conditions for the SYBR RT–PCR (Perfect Real Time) were as follows: an initial step of 15 min at 42 °C and 2 min at 95 °C and then 40 amplification cycles of denaturation at 95 °C for 15 s and annealing and extension at 60 °C for 1 min.

**Table 1:** Primer pairs and probes used for real-time PCR.

Gene		Primer sequence (5’→3’)	Note
*α-SMA*	Forward	AAT GGC TCT GGG CTC TGT AA	SYBR Green
	Reverse	TCT CTT GCT CTG GGC TTC AT	
*Col (1)*	Forward	ACG TCC TGG TGA AGT TGG TC	SYBR Green
	Reverse	TCC AGC AAT ACC CTG AGG TC	
*Col (4)*	Forward	TTT CCA GGG TTA CAA GGT GT	SYBR Green
	Reverse	AGT CCA GGT TCT CCA GCA TC	
*TGF-β1*	Forward	ATT CCT GGC GTT ACC TTG G	SYBR Green
	Reverse	AGC CCT GTA TTC CGT CTC CT	
*TNF-α*	Forward	GAC GTG GAA CTG GCA GAA GAG	SYBR Green
	Reverse	TTG GTG GTT TGT GAG TGT GAG	
*CK7*	Forward	AGG AAC AGA AGT CAG CCA AGA G	SYBR Green
	Reverse	GCA ACA CAA ACT CAT TCT CAG C	
*CK19*	Forward	GAT CTG CGT AGT GTG G-3′	SYBR Green
	Reverse	AAA ACC AAA CTG GGG ATG-3′	
*Numb*	Forward	GCT ACT TTC GAT GCC AGT AGA ACC A	SYBR Green
	Reverse	CTG TTG CCA GGA GCC ACT GA	
*RBP-Jκ*	Forward	TTG CTT ACC TTC AGG CGT GTG	SYBR Green
	Reverse	GCC CAA TGA GTC TGC TGC AA	
*Hes1*	Forward	GAC GGC CAA TTT GCT TTC	SYBR Green
	Reverse	GAC ACT GCG TTA GGA CCC	
*Notch1*	Forward	TGG ATG AGG AAG ACA AGC ATT A	SYBR Green
	Reverse	GAA AAG CCA CCG AGA TAG TCA G	
*Notch2*	Forward	GAG GAA GAA GTG TCT CAA	SYBR Green
	Reverse	GTG GCA TCA GAA ACA TAT G	
*Notch3*	Forward	GAC AAG GAC CAC TCC CAC TACT	SYBR Green
	Reverse	ATC CAC ATC ATC CTC ACA ACT G	
*Notch4*	Forward	TGT CAG GAA CCA GTG TCA GAA C	SYBR Green
	Reverse	CCT GGG CTT CAC ATT CAT CTA T	
*JAG1*	Forward	CCA TCA AGG ATT ATG AGA AC	SYBR Green
	Reverse	TGG TGC TTA TCC ATA TCA	
*JAG2*	Forward	AAA TGA GTG GTC CGT GGC AGA	SYBR Green
	Reverse	TGG TTG GAA GCC TTG TCT GCT	
*DLL1*	Forward	GTG TGC AGA TGG TCC TTG CTT C	SYBR Green
	Reverse	CTG ACA TCG GCA CAG GTA GGA G	
*DLL4*	Forward	GCA GAA CCA CAC ACT GGA CTA T	SYBR Green
	Reverse	TGG CAC CTT CTC TCC TAA ACT C	
*Sox9*	Forward	GAA AGA CCA CCC CGA TTA CAA G	SYBR Green
	Reverse	AAG ATG GCG TTA GGA GAG ATG TG	
*EpCam*	Forward	TGT GGA CAT AGC TGA TGT GGC TTA C	SYBR Green
	Reverse	CAC CCT CAG GTC CAT GCT CTT A	
*LNX1*	Forward	TGC TGC CAG GAG ACA TCA T	SYBR Green
	Reverse	CAT TGC TTC TGC TAC GGA ACT T	
*ITCH*	Forward	ATG GGA GAT TTG TCA GTT TGT C	SYBR Green
	Reverse	CAG CGT CAT TCT GTG TAG CA	
*ALB*	Forward	AAG GCA CCC CGA TTA CTC CG	SYBR Green
	Reverse	TGC GAA GTC ACC CAT CAC CG	
*HNF4α*	Forward	CGG GCC ACT GGC AAA CAC	SYBR Green
	Reverse	GTA ATC CTC CAG GCT CAC	
*Desmin*	Forward	CAATGTGAAGATGGCCTTGGATG	SYBR Green
	Reverse	AAGTTGAGAGCAGAGAAGGTCTG	
*GAPDH*	Forward	GGC ACA GTC AAG GCT GAG AAT G	SYBR Green
	Reverse	ATG GTG GTG AAG ACG CCA GTA	

### Immunoblot analysis

Liver tissue was lysed in RIPA buffer containing a mixture of protease inhibitors and phosphatase inhibitors and then homogenized in ice-cold water. Protein concentrations were determined using a BCA protein assay kit (Thermo). Total proteins were resolved on SDS–PAGE gels, transferred onto PVDF membranes, and blocked with a 5% (w/v) bovine serum albumin (Gibco) solution. The following dilutions of primary antibodies were used: α-SMA (1:1,000), TNF-α (1:1,000), Numb (1:300), RBP-Jκ (1:1,000), Hes1 (1:500), CK7 (1:1,500), CK19 (1:1,000), Sox9 (1:2,000), EpCam (1:1,000), HNF4α (1:1,000), Desmin (1:500) and GAPDH (1:10,000). The following secondary antibodies were used: IRDye 800CW-conjugated donkey to mouse IgG (H + L) (1:10,000) and IRDye 680RD-conjugated donkey to rabbit IgG (H + L) (1:1,000). Finally, the data were analyzed using Odyssey 2.1 software.

### Statistical analysis

All data are presented as the means ± SD. Statistical analyses of multiple groups were performed using analysis of variance (ANOVA) with SPSS 24.0 software, and *P* < 0.05 was considered statistically significant.

## Supplementary Information

Below is the link to the electronic supplementary material.

Supplementary Material 1

## Data Availability

All data generated or analysed during this study are included in this published article and its supplementary information files.
