# Supplementary material for: Numb-exon3 and full length Numb equivalently alleviate cholestatic liver fibrosis by inhibiting ductular reaction
Source: Sci Rep. 2025 Nov 14;15:39983. doi: 10.1038/s41598-025-23696-3 (PMC12618559; doi:10.1038/s41598-025-23696-3)

Supplementary Materials for

***Numb*-exon3 and full length *Numb* equivalently alleviate cholestatic liver fibrosis by inhibiting ductular reaction**

Yan-nan Xu^#^, Meng-yao Zong^#^, Wen Xu, Shi-hao Zhang, Dan-yang Wang, Xin-rui Zheng, Fei-fei Xing, Jun-yi Zhan, Jia-mei Chen, Gao-feng Chen, Ping Liu^§^, Wei Liu^§^, Yong-ping Mu^§^

Correspondence to: [ypmu8888@126.com,](mailto:ypmu8888@126.com,) Liuliver@vip.sina.com and lwhzayl@163.com

**Figure. S1**


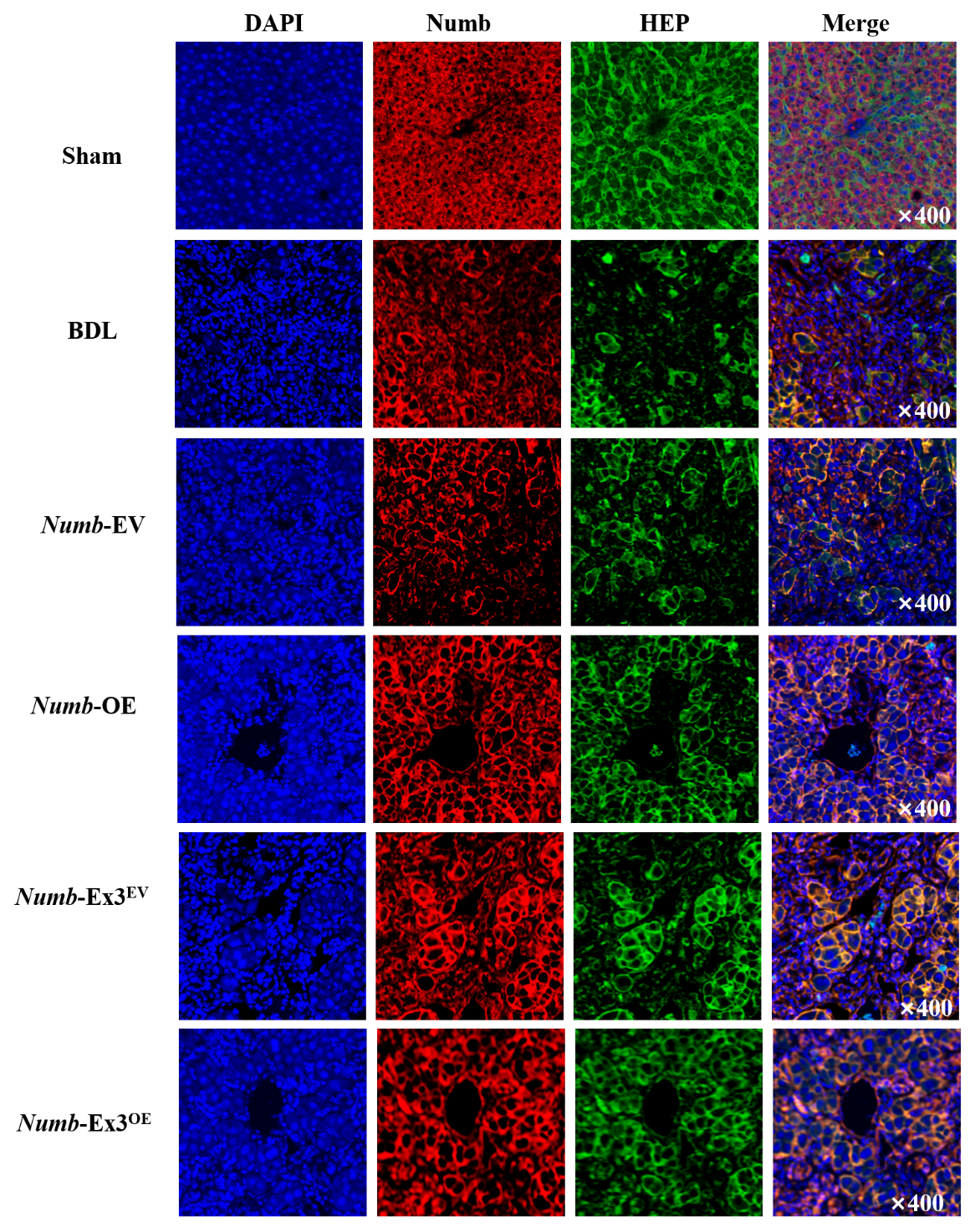


**Suppl. Fig. 1. The Numb and HEP immunofluorescence co-staining** .

Sham, sham group; BDL, bile duct ligation group; *Numb*-EV, *Numb*-Empty vector group; *Numb*-OE, *Numb*-overexpression group. *Numb*-EX3^EV^, *Numb*-Exon3^Empty vector^ group; *Numb*-EX3^OE^, *Numb*-Exon3^overexpression^ group.

**Figure. S2**


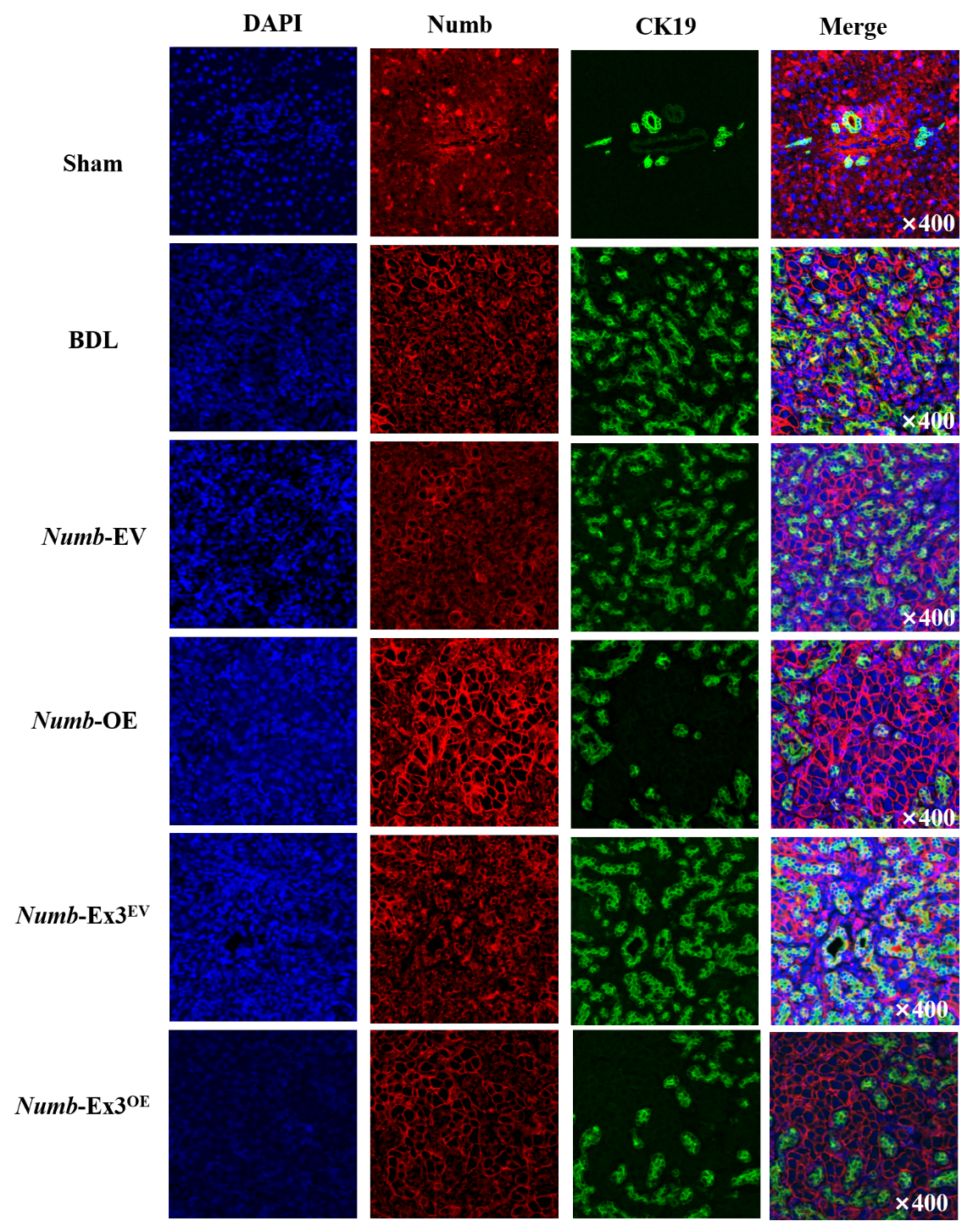


**Suppl. Fig. 2. The Numb and CK19 immunofluorescence co-staining**

Sham, sham group; BDL, bile duct ligation group; *Numb*-EV, *Numb*-Empty vector group; *Numb*-OE, *Numb*-overexpression group. *Numb*-EX3^EV^, *Numb*-Exon3^Empty vector^ group; *Numb*-EX3^OE^, *Numb*-Exon3^overexpression^ group.

**Figure. S3**


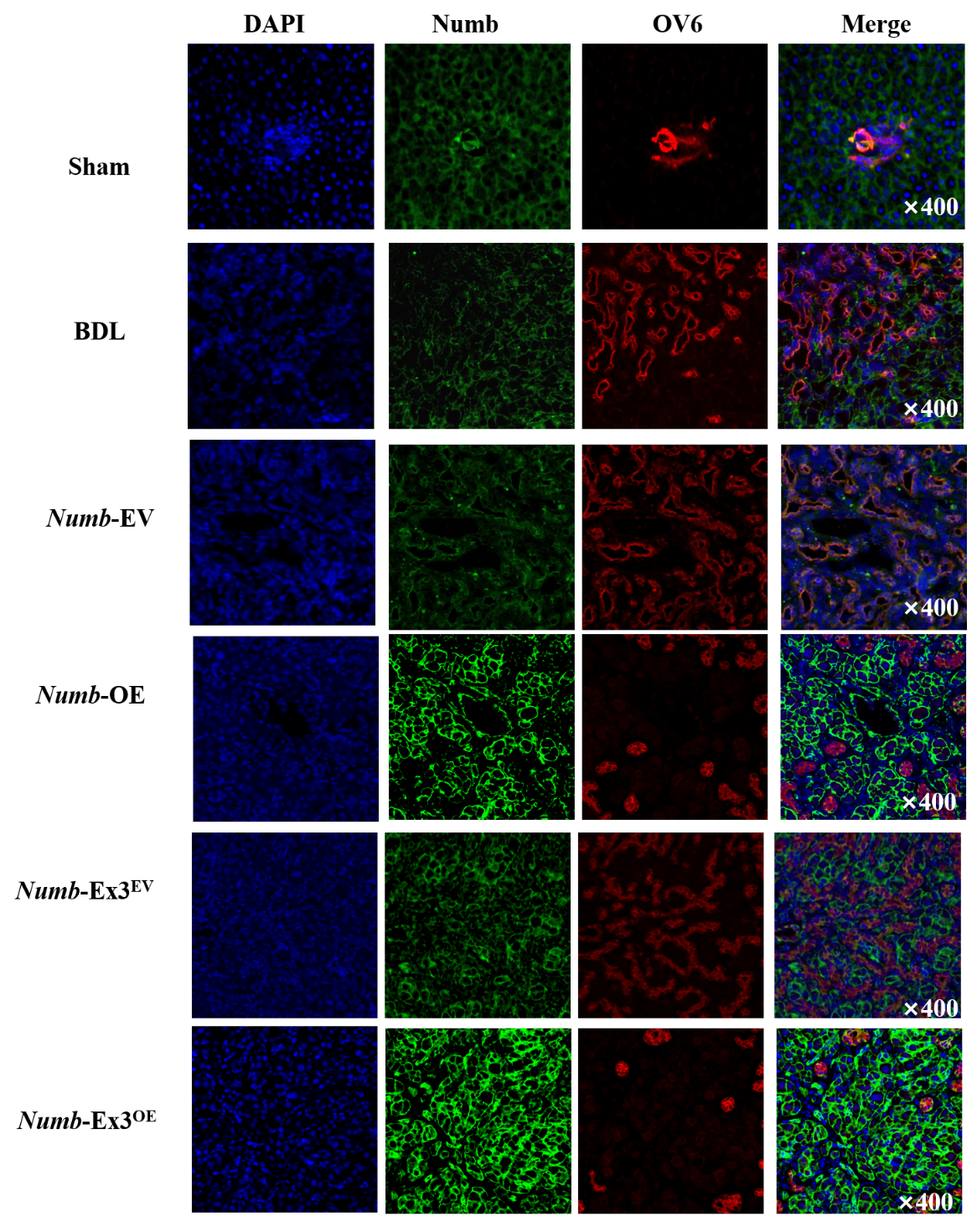


**Suppl. Fig. 3. The Numb and OV6 immunofluorescence co-staining**.

Sham, sham group; BDL, bile duct ligation group; *Numb*-EV, *Numb*-Empty vector group; *Numb*-OE, *Numb*-overexpression group. *Numb*-EX3^EV^, *Numb*-Exon3^Empty vector^ group; *Numb*-EX3^OE^, *Numb*-Exon3^overexpression^ group.

**Figure. S4**


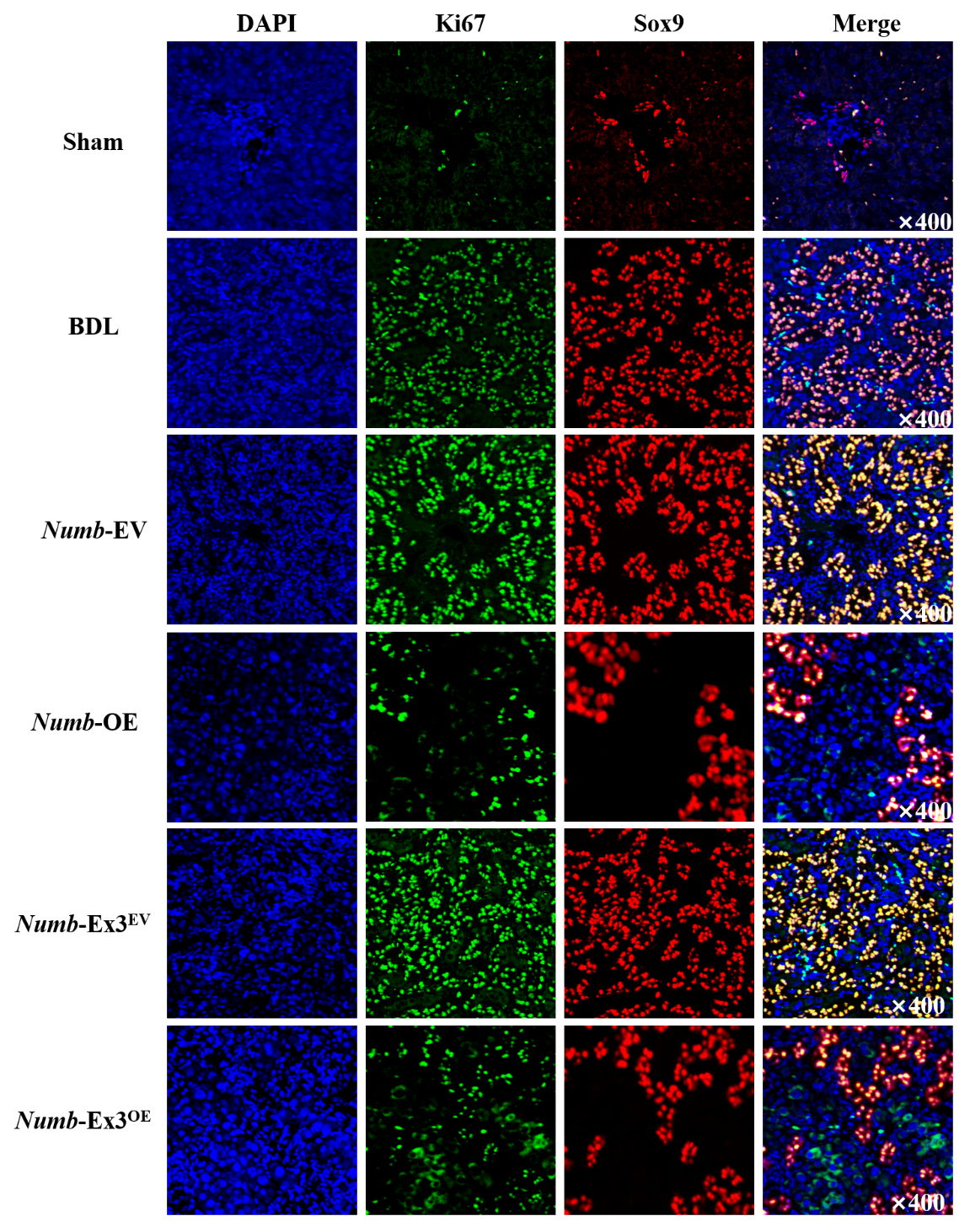


**Suppl. Fig. 4. The Ki67 and Sox9 immunofluorescence co-staining**.

Sham, sham group; BDL, bile duct ligation group; *Numb*-EV, *Numb*-Empty vector group; *Numb*-OE, *Numb*-overexpression group. *Numb*-EX3^EV^, *Numb*-Exon3^Empty vector^ group; *Numb*-EX3^OE^, *Numb*-Exon3^overexpression^ group.

**Figure. S5**


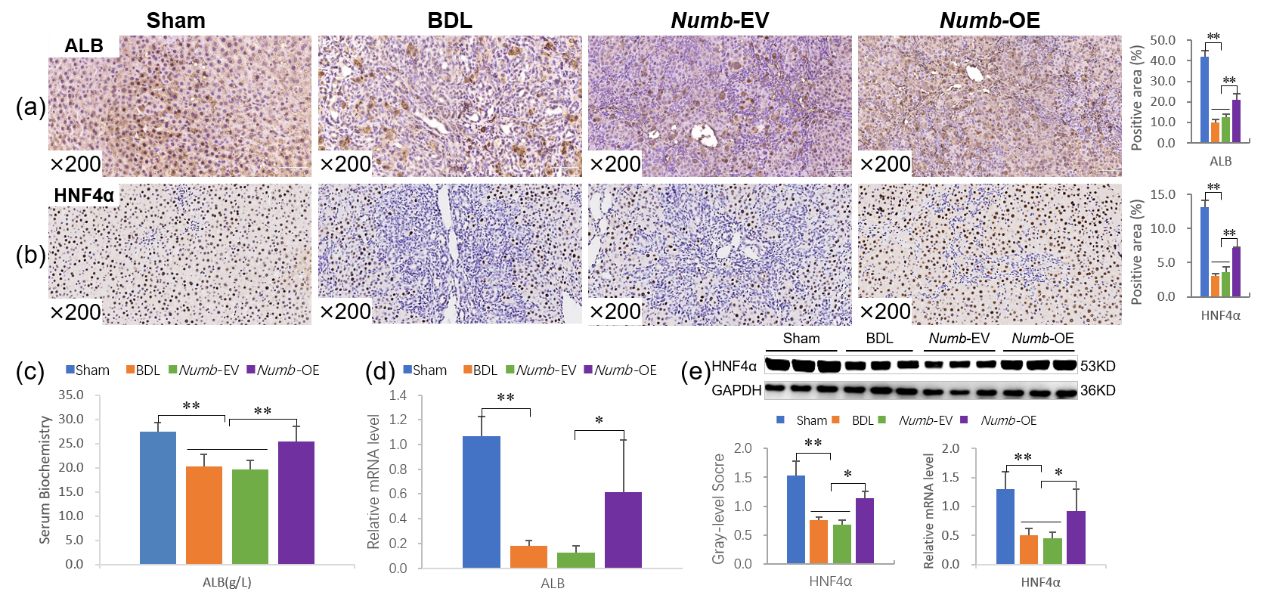


**Suppl. Fig. 5. The *Numb* gene promotes hepatocyte regeneration**. (a) ALB immunostaining (×200) and its positive area (%). (b) HNF4α immunostaining (×200) and its positive area (%). (c) Serum ALB content (g/L). (d) The expression level of ALB mRNA in liver. (e) HNF4α immunoblotting band, gray‑level integration and mRNA expression (*n* = 6/per group). * *P* <0.05, ** *P* <0.01. Sham, sham group; BDL, bile duct ligation group; *Numb*-EV, *Numb*-Empty vector group; *Numb*-OE, *Numb*-overexpression group.

**Supplementary Text 1. The target sequence of AAV8.*Numb***

AATATTCACCAGCAGCCTCCCCCGTTGCCCCTCTGGATCCACTGCTTAAATACGGACGAGGACAGGGCCCTGTCTCCTCAGCTTCAGGCACCACCACTGACCTGGGACAGTGAATACCGGTCGCCACCATGAACAAACTACGGCAGAGTTTCAGGAGAAAGAAAGATGTTTACGTCCCAGAGGCCAGCCGTCCACATCAGTGGCAGACAGATGAAGAGGGAGTCCGCACTGGAAAGTGCAGCTTCCCAGTTAAGTACCTTGGCCACGTAGAGGTTGATGAGTCAAGAGGAATGCACATCTGTGAAGATGCCGTCAAAAGATTGAAAGCTGAAAGGAAGTTCTTCAAAGGCTTCTTTGGAAAAACGGGAAAGAAGGCCGTCAAAGCTGTCCTGTGGGTATCCGCCGACGGGCTGAGGGTCGTGGATGAAAAAACTAAGGACCTCATAGTTGACCAGACAATAGAAAAAGTTTCTTTCTGCGCCCCCGATAGGAACTTTGACAGAGCCTTTTCTTACATATGTCGAGATGGCACCACTCGGCGATGGATCTGTCATTGCTTCATGGCTGTCAAAGACACGGGGGAAAGACTGAGCCATGCCGTGGGCTGTGCTTTTGCAGCCTGTTTAGAGCGTAAACAGAAGCGGGAAAAGGAGTGTGGAGTCACTGCTACTTTCGATGCCAGTAGAACCACTTTTACAAGAGAAGGATCATTCCGTGTCACAACTGCCACGGAACAAGCTGAAAGAGAGGAGATCATGAAACAGTTGCAAGATGCCAAGAAAGCTGAGACAGATAAGACCGTTGGTCCATCAGTGGCTCCTGGCAACAGTGCTCCATCGCCGTCCTCTCCCACCTCCCCAACTCTGGATCCCACTGCTTCTTTAGAGATGAACAATCCTCATGCTATCCCACGCCGGCATGCACCAATTGAACAGCTTGCTCGCCAAGGCTCTTTCCGGGGATTTCCTGCTCTTAGCCAGAAGATGTCACCCTTTAAACGCCAGCTGTCCCTACGCATCAATGAGCTGCCTTCCACTATGCAGAGGAAGACTGATTTCCCAATAAAAAACACAGTGCCTGAGGTGGAAGGAGAGGCAGAAAGCATCAGCTCCCTGTGCTCCCAGATCACCAGTGCCTTCAGCACACCCTGTGAGGACCCCTTCTCCTCTGCCCCAATGACCAAACCAGTGACATTAGTGGCACCACAGTCTCCTGTGTTACAAGCTAATGGCACTGACTCAGCCCTCCATGTGCTTACCGCAAAGCCAGCCAGTACTGCTCTAGCACCCGTAGCAATGCCTGTCCGTGAAACCAACCCTTGGGCCCATGCCCCTGATGCTGCTAACAAGGAAATTGCAGCCATACATTCGGGGACTGAGTGGGGTCAGTCTTCTGGTGCTGCCTCTCCAGGTCTCTTCCAGGCTGGTCACAGACGCACTCCCTCTGAGGCTGACCGTTGGTTAGAAGAAGTATCAAAAAGTGTGCGGGCCCAGCAGCCGCAGGTCTCAGCCGCCCCTCTGCAGCCAGTTCTGCAGCCTCCTCCGCCCGCTGCCATTGCCCCTCCAGCACCTCCTTTCCAAGGACATGCATTCCTCACTTCTCAGCCTGTGCCAGTGGGTGTGGTCCCACCCCTACAACCAGCCTTTGTCTCTACCCAGTCCTACCCTGTGGCCAATGGGATGCCCTATCCAGCCTCTAATGTGCCTGTAGTGGGCATCACCCCATCCCAGATGGTAGCCAATGTGTTTGGCACTGCAGGCCATCCTCAGGCCACTCATCCACATCAGTCCCCAAGCCTGGCCAAGCAGCAGACATTCCCTCAATATGAGACAAGTAGTGCTACCACCAGTCCCTTCTTTAAGCCTTCTGCTCAGCACCTCAATGGTTCTGCAGCTTTCAATGGTGTAGACAATAGCGGGCTAGTCTCAGGAAACAGACCTGCACAAGTCCCTCCAGGCACCTGCCCAGTGGATCCTTTTGAGGCCCAGTGGGCTGCACTAGAAAGCAAGCCCAAGCAGCGCACCAACCCCTCTCCTACCAACCCTTTCTCCAGTGATGCACAGAAGGCATTTGAAATAGAGCTTTCGAGGGATCCCGCCACCATGGTGAGCAAGGGCGAGGAGCTGTTCACCGGGGTGGTGCCCATCCTGGTCGAGCTGGACGGCGACGT

**Supplementary Text 2. The target sequence of AAV8.*Numb*-Exon3**

ACGGACGAGGACAGGGCCCTGTCTCCTCAGCTTCAGGCACCACCACTGACCTGGGACAGTGAATACCGGTCGCCACCATGAACAAACTACGGCAGAGTTTCAGGAGAAAGAAAGATGTTTACGTCCCAGAGGCCAGCCGTCCACATCAGTGGCAGACAGATGAAGAGGGAGTCCGCACTGGAAAGTGCAGCTTCCCAGTTAAGTACCTTGGCCACGTAGAGGTTGATGAGTCAAGAGGAATGCACATCTGTGAAGATGCCGTCAAAAGATTGAAAGCTGAAAGGAAGTTCTTCAAAGGCTTCTTTGGAAAAACGGGAAAGAAGGCCGTCAAAGCTGTCCTGTGGGTATCCGCCGACGGGCTGAGGGTCGTGGATGAAAAAACTAAGGACCTCATAGTTGACCAGACAATAGAAAAAGTTTCTTTCTGCGCCCCCGATAGGAACTTTGACAGAGCCTTTTCTTACATATGTCGAGATGGCACCACTCGGCGATGGATCTGTCATTGCTTCATGGCTGTCAAAGACACGGGGGAAAGACTGAGCCATGCCGTGGGCTGTGCTTTTGCAGCCTGTTTAGAGCGTAAACAGAAGCGGGAAAAGGAGTGTGGAGTCACTGCTACTTTCGATGCCAGTAGAACCACTTTTACAAGAGAAGGATCATTCCGTGTCACAACTGCCACGGAACAAGCTGAAAGAGAGGAGATCATGAAACAGTTGCAAGATGCCAAGAAAGCTGAGACAGATAAGACCGTTGGTCCATCAGTGGCTCCTGGCAACAGTGCTCCATCGCCGTCCTCTCCCACCTCCCCAACTCTGGATCCCACTGCTTCTTTAGAGATGAACAATCCTCATGCTATCCCACGCCGGCATGCACCAATTGAACAGCTTGCTCGCCAAGGCTCTTTCCGGGGATTTCCTGCTCTTAGCCAGAAGATGTCACCCTTTAAACGCCAGCTGTCCCTACGCATCAATGAGCTGCCTTCCACTATGCAGAGGAAGACTGATTTCCCAATAAAAAACACAGTGCCTGAGGTGGAAGGAGAGGCAGAAAGCATCAGCTCCCTGTGCTCCCAGATCACCAGTGCCTTCAGCACACCCTGTGAGGACCCCTTCTCCTCTGCCCCAATGACCAAACCAGTGACATTAGTGGCACCACAGTCTCCTGTGTTACAAGGGACTGAGTGGGGTCAGTCTTCTGGTGCTGCCTCTCCAGGTCTCTTCCAGGCTGGTCACAGACGCACTCCCTCTGAGGCTGACCGTTGGTTAGAAGAAGTATCAAAAAGTGTGCGGGCCCAGCAGCCGCAGGTCTCAGCCGCCCCTCTGCAGCCAGTTCTGCAGCCTCCTCCGCCCGCTGCCATTGCCCCTCCAGCACCTCCTTTCCAAGGACATGCATTCCTCACTTCTCAGCCTGTGCCAGTGGGTGTGGTCCCACCCCTACAACCAGCCTTTGTCTCTACCCAGTCCTACCCTGTGGCCAATGGGATGCCCTATCCAGCCTCTAATGTGCCTGTAGTGGGCATCACCCCATCCCAGATGGTAGCCAATGTGTTTGGCACTGCAGGCCATCCTCAGGCCACTCATCCACATCAGTCCCCAAGCCTGGCCAAGCAGCAGACATTCCCTCAATATGAGACAAGTAGTGCTACCACCAGTCCCTTCTTTAAGCCTTCTGCTCAGCACCTCAATGGTTCTGCAGCTTTCAATGGTGTAGACAATAGCGGGCTAGTCTCAGGAAACAGACCTGCACAAGTCCCTCCAGGCACCTGCCCAGTGGATCCTTTTGAGGCCCAGTGGGCTGCACTAGAAAGCAAGCCCAAGCAGCGCACCAACCCCTCTCCTACCAACCCTTTCTCCAGTGATGCACAGAAGGCATTTGAAATAGAGCTTGTCTCGAGGGATCCCGCCACCATGGTGAGCAAGGGCGAGGAGCTGTTCACCGGGGTGGTGCCCATCCTGGTCGAGCTGGACGGCGACGTAAACGGCCACAAGTTCAGCGTGTCCGGCGAGGGCGAGGGCGATGCCACCTACGGCAAGCTGACCCTGAAGTTCATCTGCACCACCGGCAAG

**Supplementary Text 3. The target sequence of LV-*Numb***

ATGAACAAACTACGGCAGAGTTTCAGGAGAAAGAAAGATGTTTACGTCCCAGAGGCCAGCCGTCCACATCAGTGGCAGACAGATGAAGAGGGAGTCCGCACTGGAAAGTGCAGCTTCCCAGTTAAGTACCTTGGCCACGTAGAGGTTGATGAGTCAAGAGGAATGCACATCTGTGAAGATGCCGTCAAAAGATTGAAAGCTGAAAGGAAGTTCTTCAAAGGCTTCTTTGGAAAAACGGGAAAGAAGGCCGTCAAAGCTGTCCTGTGGGTATCCGCCGACGGGCTGAGGGTCGTGGATGAAAAAACTAAGGACCTCATAGTTGACCAGACAATAGAAAAAGTTTCTTTCTGCGCCCCCGATAGGAACTTTGACAGAGCCTTTTCTTACATATGTCGAGATGGCACCACTCGGCGATGGATCTGTCATTGCTTCATGGCTGTCAAAGACACGGGGGAAAGACTGAGCCATGCCGTGGGCTGTGCTTTTGCAGCCTGTTTAGAGCGTAAACAGAAGCGGGAAAAGGAGTGTGGAGTCACTGCTACTTTCGATGCCAGTAGAACCACTTTTACAAGAGAAGGATCATTCCGTGTCACAACTGCCACGGAACAAGCTGAAAGAGAGGAGATCATGAAACAGTTGCAAGATGCCAAGAAAGCTGAGACAGATAAGACCGTTGGTCCATCAGTGGCTCCTGGCAACAGTGCTCCATCGCCGTCCTCTCCCACCTCCCCAACTCTGGATCCCACTGCTTCTTTAGAGATGAACAATCCTCATGCTATCCCACGCCGGCATGCACCAATTGAACAGCTTGCTCGCCAAGGCTCTTTCCGGGGATTTCCTGCTCTTAGCCAGAAGATGTCACCCTTTAAACGCCAGCTGTCCCTACGCATCAATGAGCTGCCTTCCACTATGCAGAGGAAGACTGATTTCCCAATAAAAAACACAGTGCCTGAGGTGGAAGGAGAGGCAGAAAGCATCAGCTCCCTGTGCTCCCAGATCACCAGTGCCTTCAGCACACCCTGTGAGGACCCCTTCTCCTCTGCCCCAATGACCAAACCAGTGACATTAGTGGCACCACAGTCTCCTGTGTTACAAGCTAATGGCACTGACTCAGCCCTCCATGTGCTTACCGCAAAGCCAGCCAGTACTGCTCTAGCACCCGTAGCAATGCCTGTCCGTGAAACCAACCCTTGGGCCCATGCCCCTGATGCTGCTAACAAGGAAATTGCAGCCATACATTCGGGGACTGAGTGGGGTCAGTCTTCTGGTGCTGCCTCTCCAGGTCTCTTCCAGGCTGGTCACAGACGCACTCCCTCTGAGGCTGACCGTTGGTTAGAAGAAGTATCAAAAAGTGTGCGGGCCCAGCAGCCGCAGGTCTCAGCCGCCCCTCTGCAGCCAGTTCTGCAGCCTCCTCCGCCCGCTGCCATTGCCCCTCCAGCACCTCCTTTCCAAGGACATGCATTCCTCACTTCTCAGCCTGTGCCAGTGGGTGTGGTCCCACCCCTACAACCAGCCTTTGTCTCTACCCAGTCCTACCCTGTGGCCAATGGGATGCCCTATCCAGCCTCTAATGTGCCTGTAGTGGGCATCACCCCATCCCAGATGGTAGCCAATGTGTTTGGCACTGCAGGCCATCCTCAGGCCACTCATCCACATCAGTCCCCAAGCCTGGCCAAGCAGCAGACATTCCCTCAATATGAGACAAGTAGTGCTACCACCAGTCCCTTCTTTAAGCCTTCTGCTCAGCACCTCAATGGTTCTGCAGCTTTCAATGGTGTAGACAATAGCGGGCTAGTCTCAGGAAACAGACCTGCACAAGTCCCTCCAGGCACCTGCCCAGTGGATCCTTTTGAGGCCCAGTGGGCTGCACTAGAAAGCAAGCCCAAGCAGCGCACCAACCCCTCTCCTACCAACCCTTTCTCCAGTGATGCACAGAAGGCATTTGAAATAGAGCTTTAG

**Supplementary Text 4. The target sequence of LV-*Numb-Exon3***

ATGAACAAACTACGGCAGAGTTTCAGGAGAAAGAAAGATGTTTACGTCCCAGAGGCCAGCCGTCCACATCAGTGGCAGACAGATGAAGAGGGAGTCCGCACTGGAAAGTGCAGCTTCCCAGTTAAGTACCTTGGCCACGTAGAGGTTGATGAGTCAAGAGGAATGCACATCTGTGAAGATGCCGTCAAAAGATTGAAAGCTGAAAGGAAGTTCTTCAAAGGCTTCTTTGGAAAAACGGGAAAGAAGGCCGTCAAAGCTGTCCTGTGGGTATCCGCCGACGGGCTGAGGGTCGTGGATGAAAAAACTAAGGACCTCATAGTTGACCAGACAATAGAAAAAGTTTCTTTCTGCGCCCCCGATAGGAACTTTGACAGAGCCTTTTCTTACATATGTCGAGATGGCACCACTCGGCGATGGATCTGTCATTGCTTCATGGCTGTCAAAGACACGGGGGAAAGACTGAGCCATGCCGTGGGCTGTGCTTTTGCAGCCTGTTTAGAGCGTAAACAGAAGCGGGAAAAGGAGTGTGGAGTCACTGCTACTTTCGATGCCAGTAGAACCACTTTTACAAGAGAAGGATCATTCCGTGTCACAACTGCCACGGAACAAGCTGAAAGAGAGGAGATCATGAAACAGTTGCAAGATGCCAAGAAAGCTGAGACAGATAAGACCGTTGGTCCATCAGTGGCTCCTGGCAACAGTGCTCCATCGCCGTCCTCTCCCACCTCCCCAACTCTGGATCCCACTGCTTCTTTAGAGATGAACAATCCTCATGCTATCCCACGCCGGCATGCACCAATTGAACAGCTTGCTCGCCAAGGCTCTTTCCGGGGATTTCCTGCTCTTAGCCAGAAGATGTCACCCTTTAAACGCCAGCTGTCCCTACGCATCAATGAGCTGCCTTCCACTATGCAGAGGAAGACTGATTTCCCAATAAAAAACACAGTGCCTGAGGTGGAAGGAGAGGCAGAAAGCATCAGCTCCCTGTGCTCCCAGATCACCAGTGCCTTCAGCACACCCTGTGAGGACCCCTTCTCCTCTGCCCCAATGACCAAACCAGTGACATTAGTGGCACCACAGTCTCCTGTGTTACAAGGGACTGAGTGGGGTCAGTCTTCTGGTGCTGCCTCTCCAGGTCTCTTCCAGGCTGGTCACAGACGCACTCCCTCTGAGGCTGACCGTTGGTTAGAAGAAGTATCAAAAAGTGTGCGGGCCCAGCAGCCGCAGGTCTCAGCCGCCCCTCTGCAGCCAGTTCTGCAGCCTCCTCCGCCCGCTGCCATTGCCCCTCCAGCACCTCCTTTCCAAGGACATGCATTCCTCACTTCTCAGCCTGTGCCAGTGGGTGTGGTCCCACCCCTACAACCAGCCTTTGTCTCTACCCAGTCCTACCCTGTGGCCAATGGGATGCCCTATCCAGCCTCTAATGTGCCTGTAGTGGGCATCACCCCATCCCAGATGGTAGCCAATGTGTTTGGCACTGCAGGCCATCCTCAGGCCACTCATCCACATCAGTCCCCAAGCCTGGCCAAGCAGCAGACATTCCCTCAATATGAGACAAGTAGTGCTACCACCAGTCCCTTCTTTAAGCCTTCTGCTCAGCACCTCAATGGTTCTGCAGCTTTCAATGGTGTAGACAATAGCGGGCTAGTCTCAGGAAACAGACCTGCACAAGTCCCTCCAGGCACCTGCCCAGTGGATCCTTTTGAGGCCCAGTGGGCTGCACTAGAAAGCAAGCCCAAGCAGCGCACCAACCCCTCTCCTACCAACCCTTTCTCCAGTGATGCACAGAAGGCATTTGAAATAGAGCTTTAG

**Original western blot images**


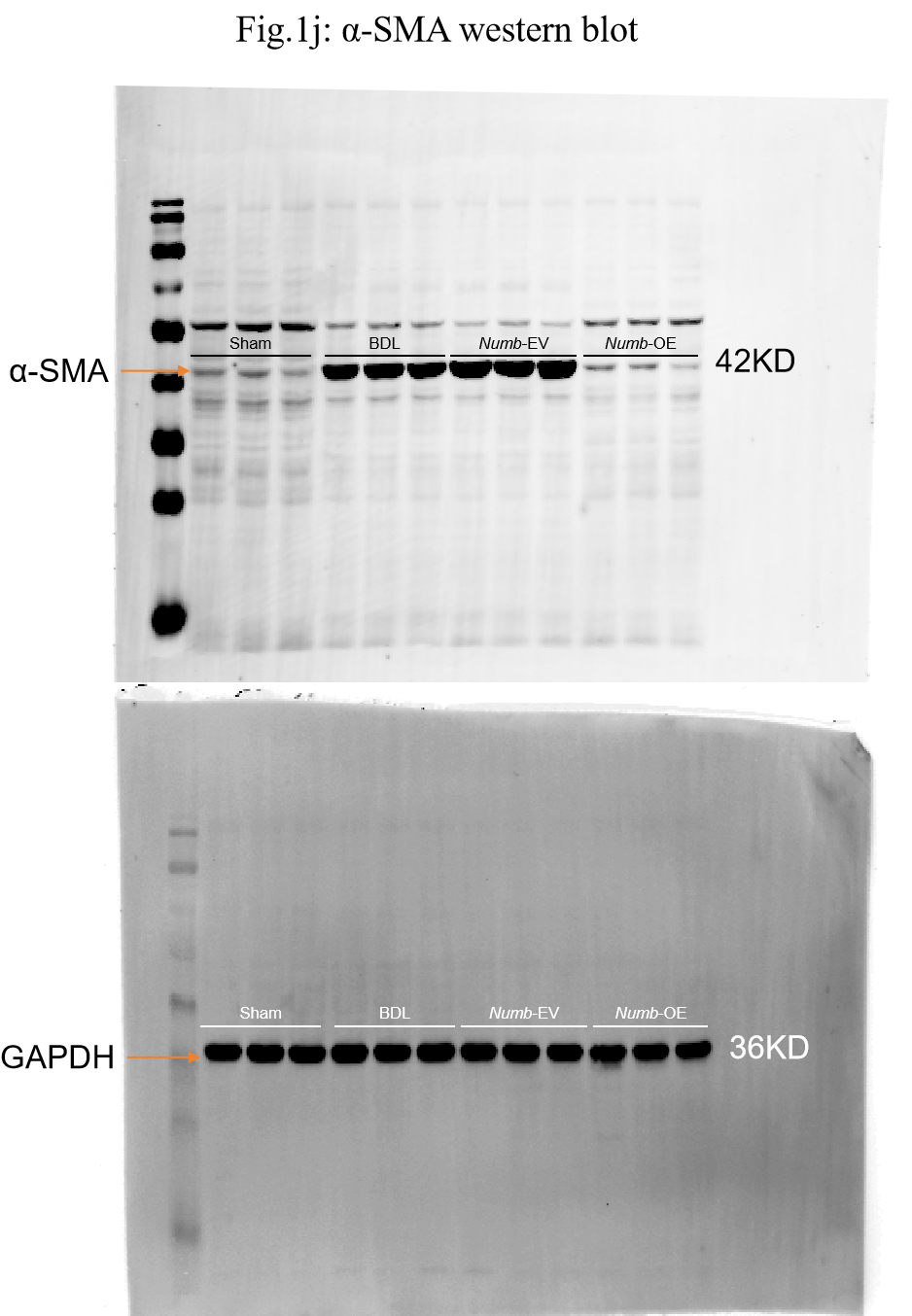


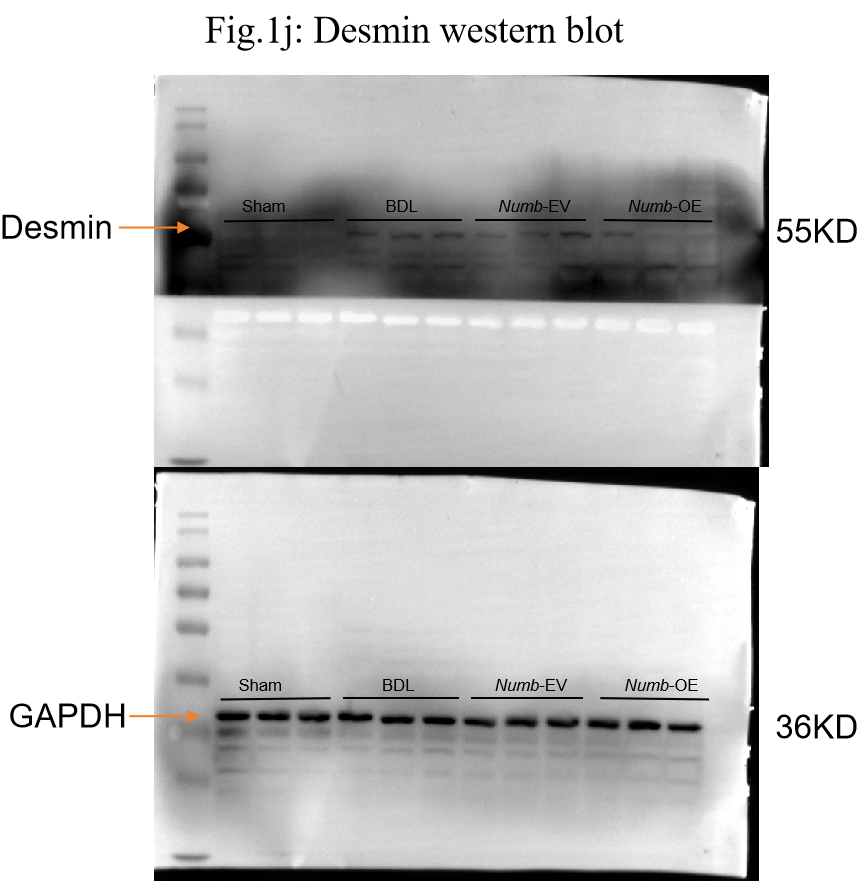


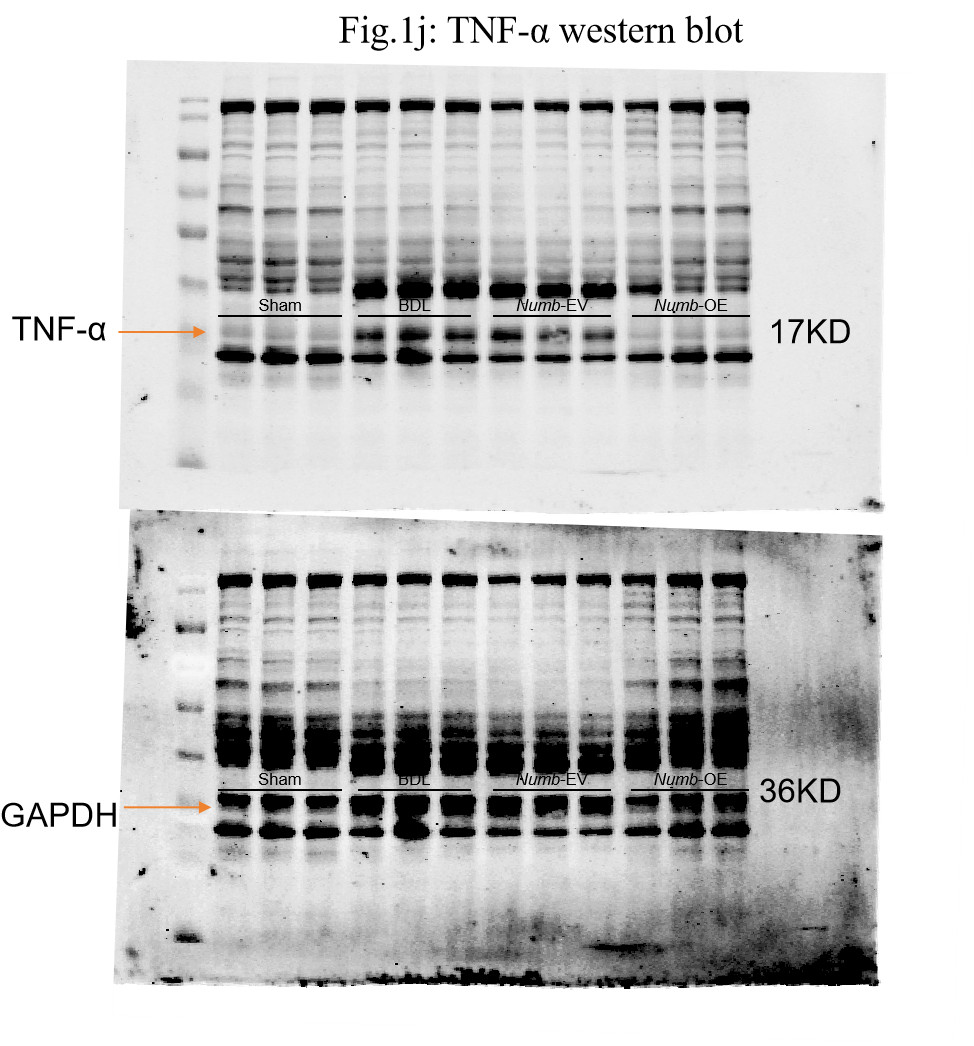


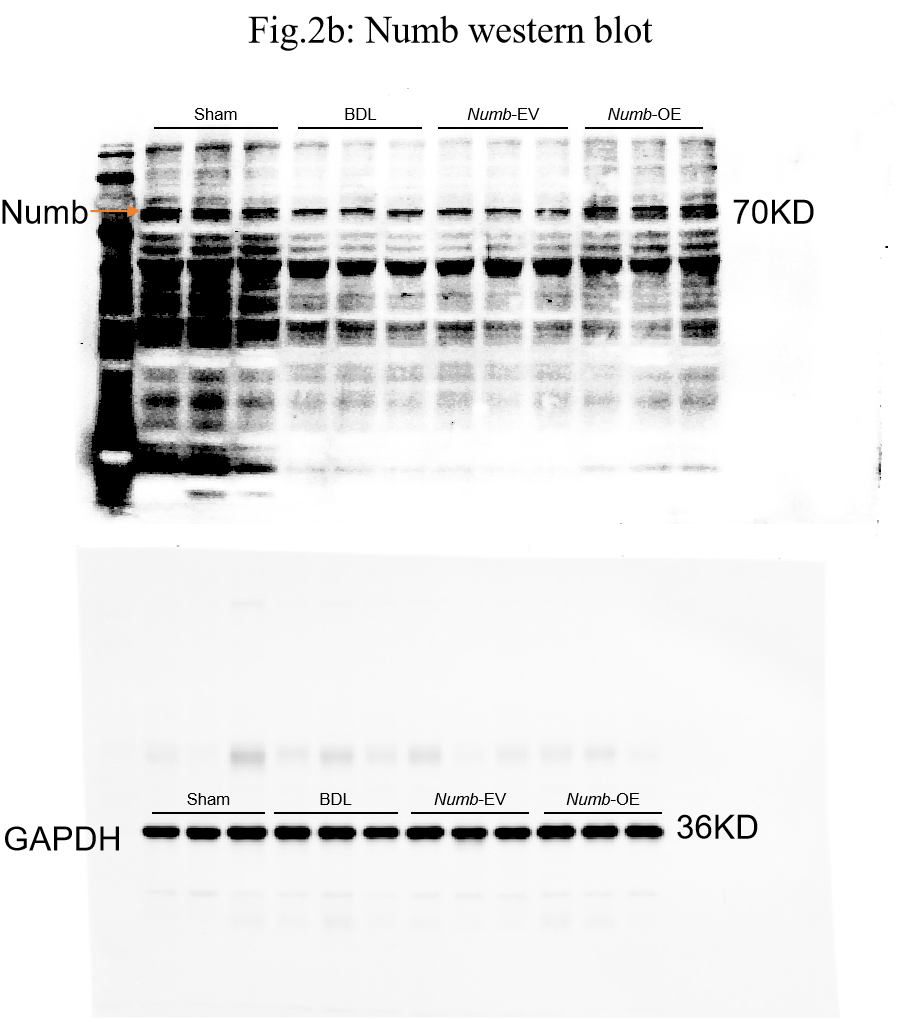


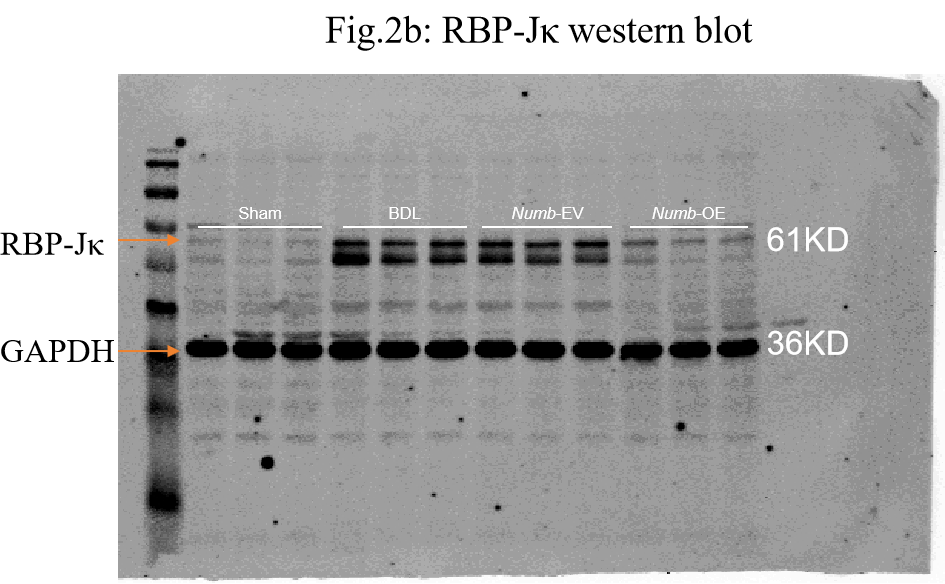


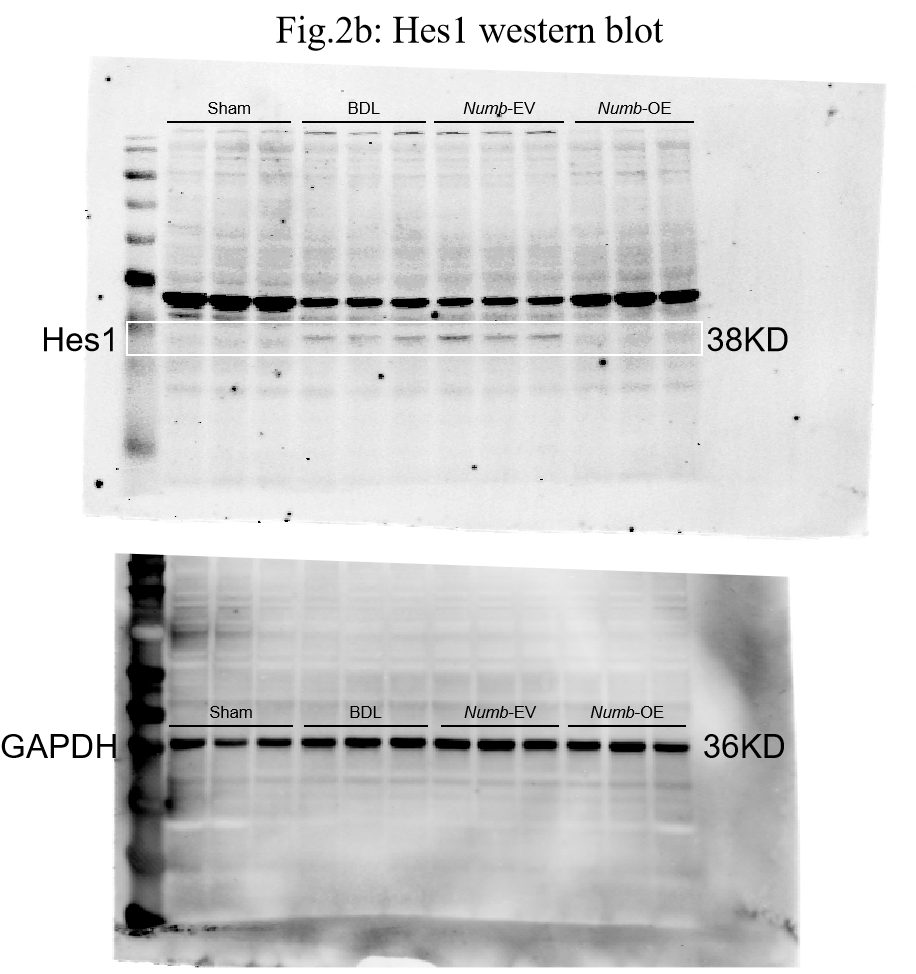


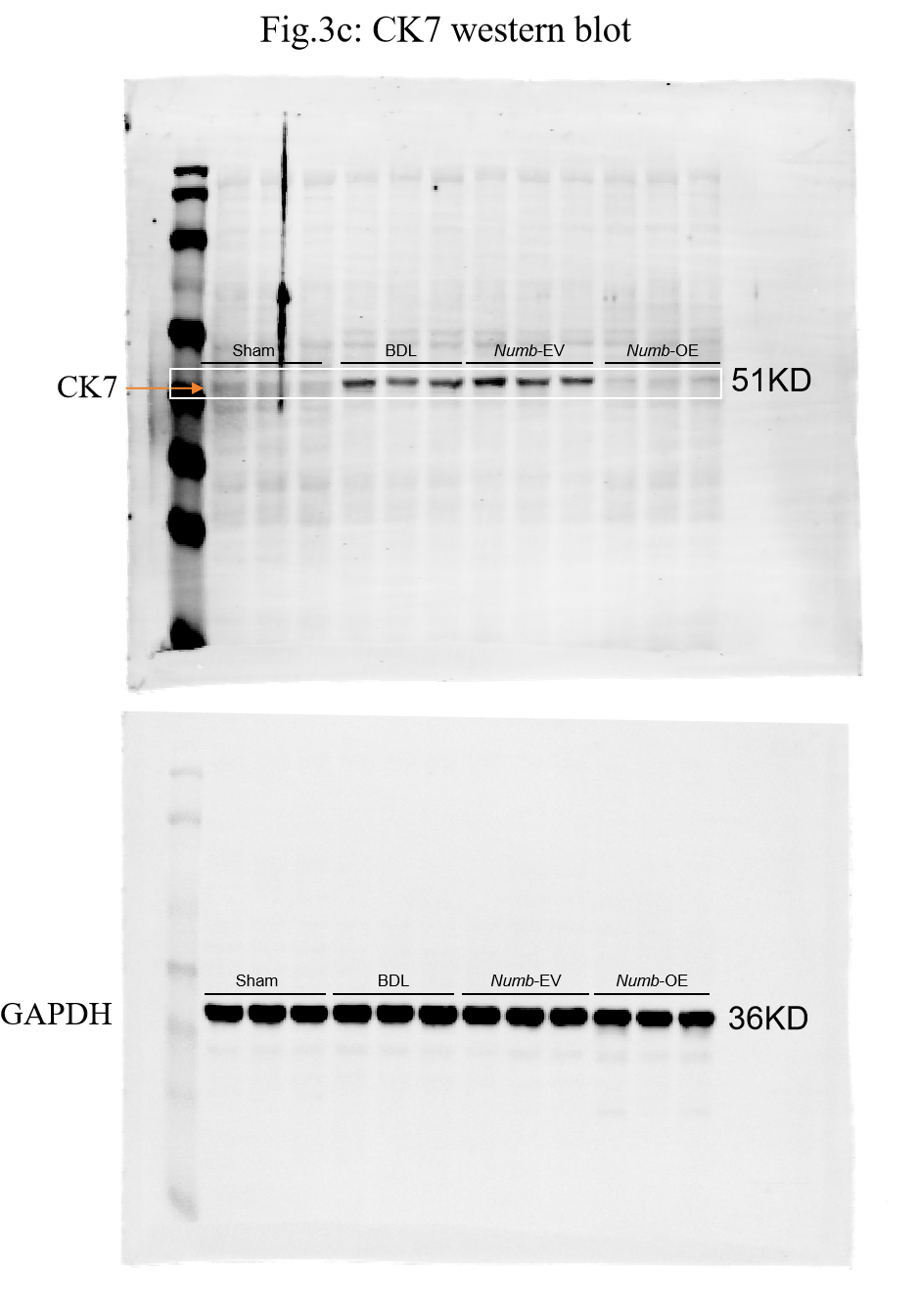


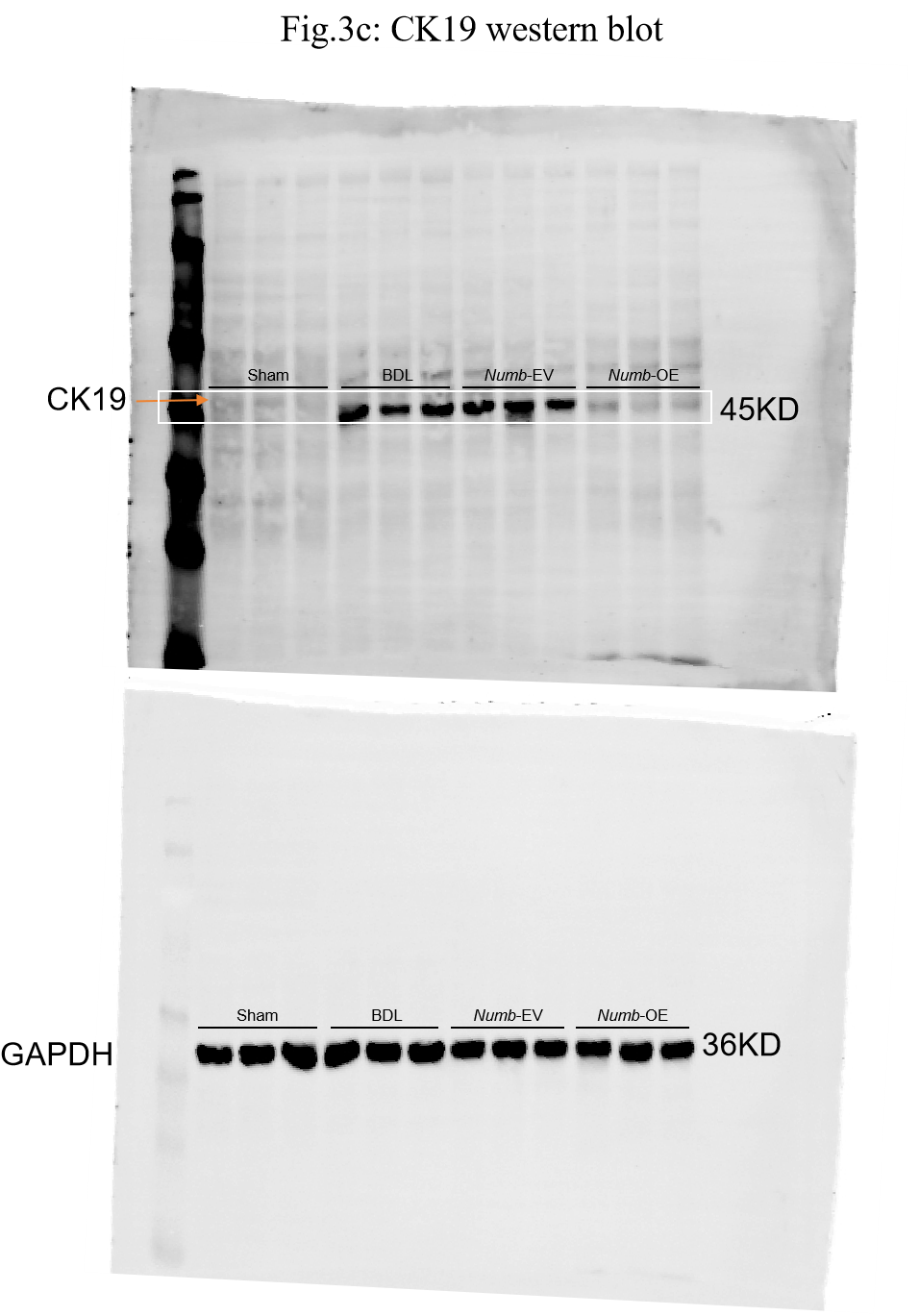


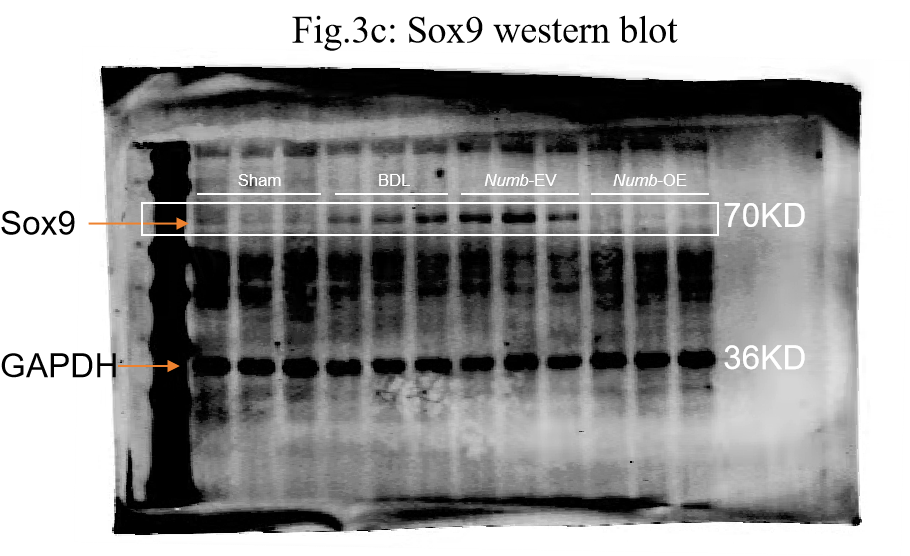


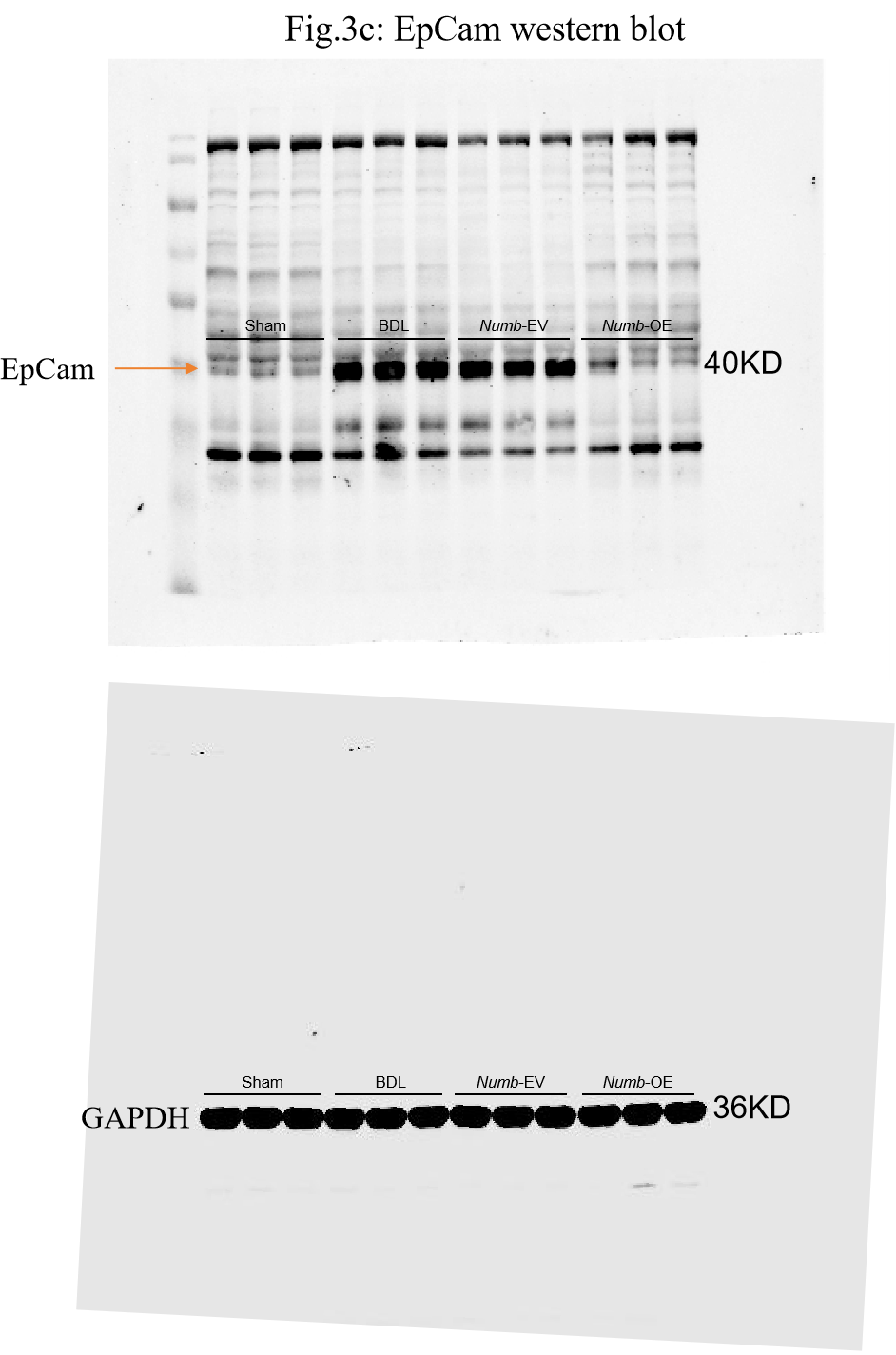


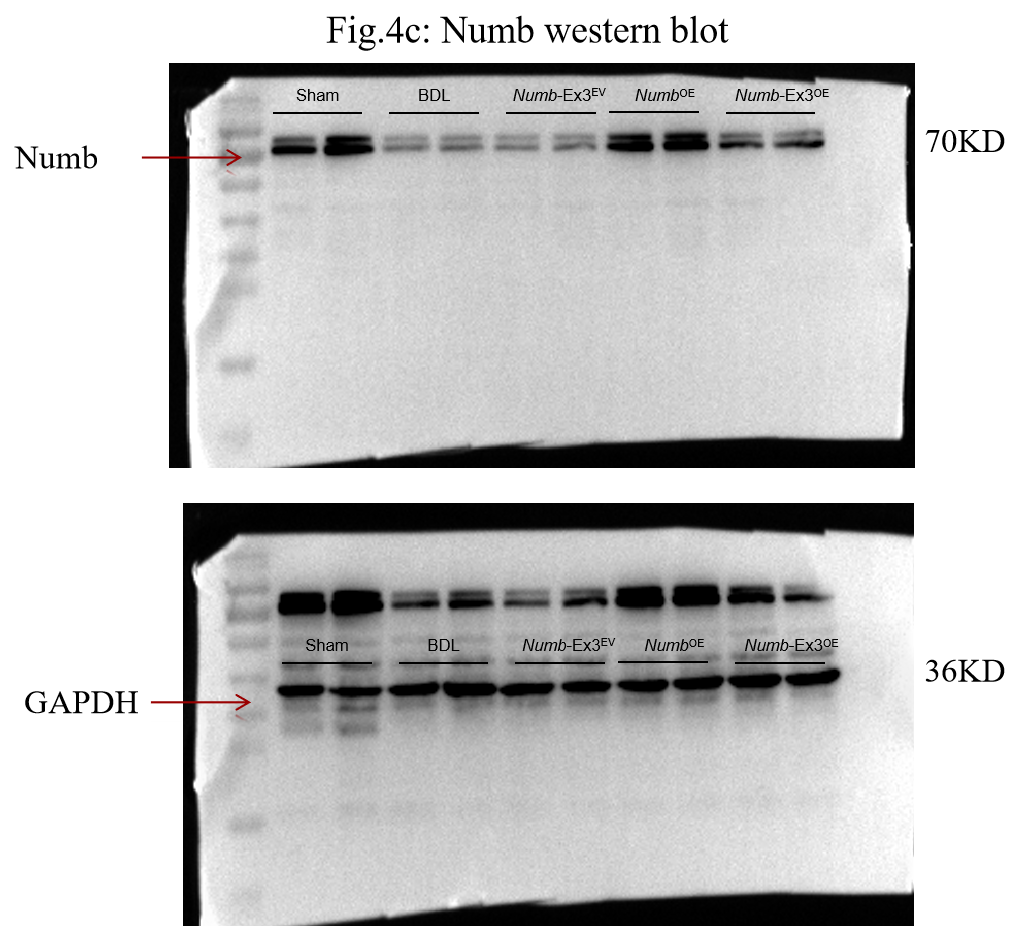


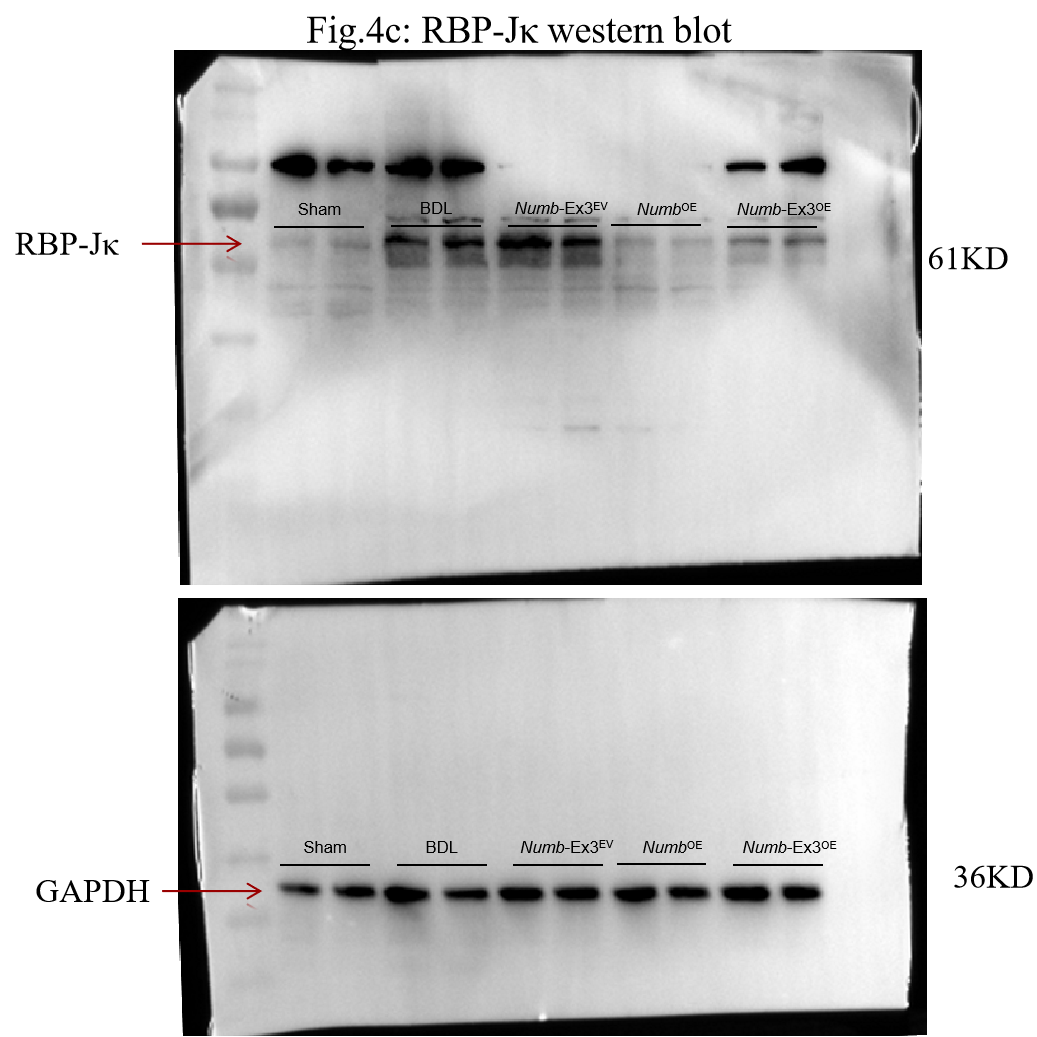


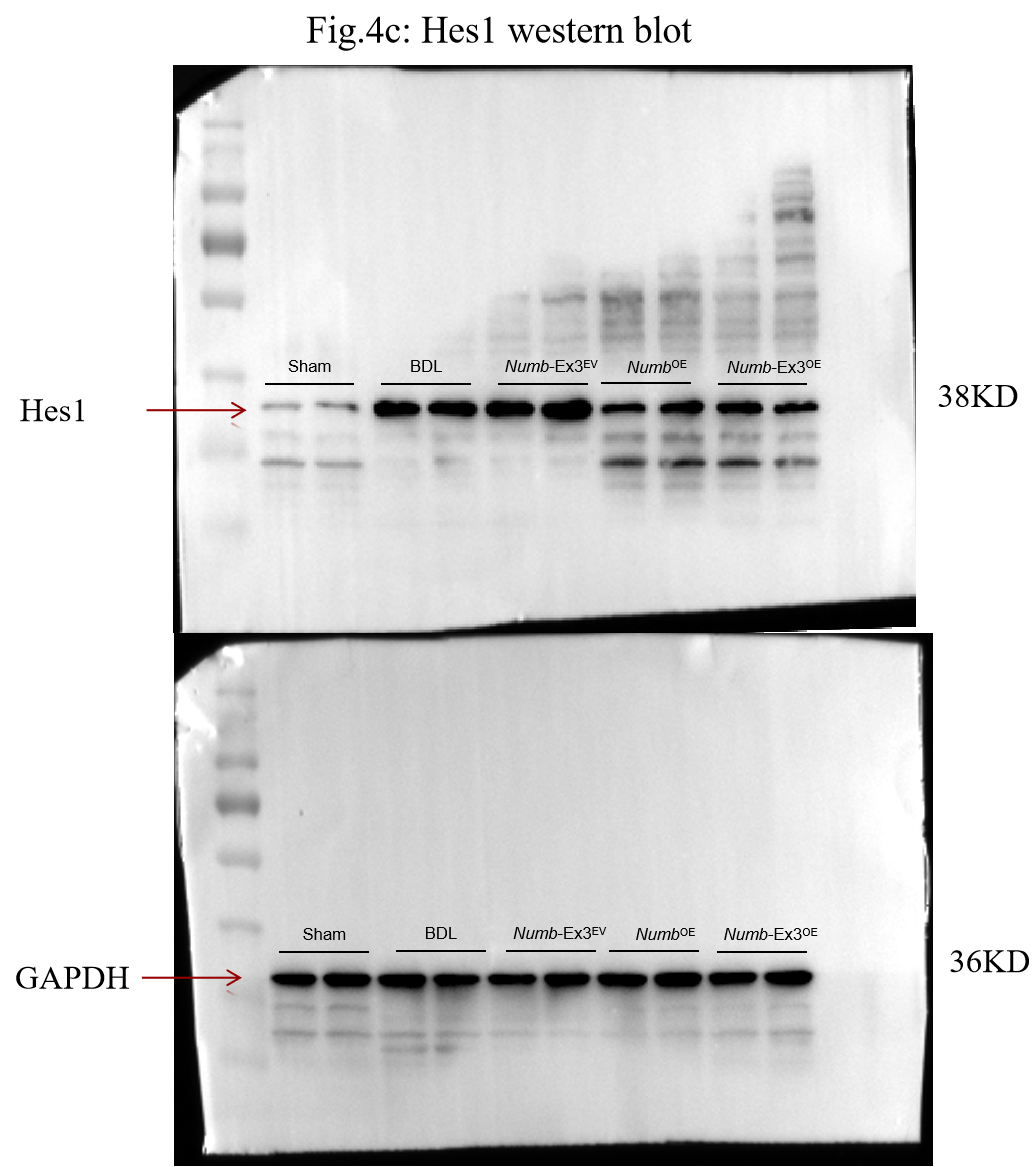


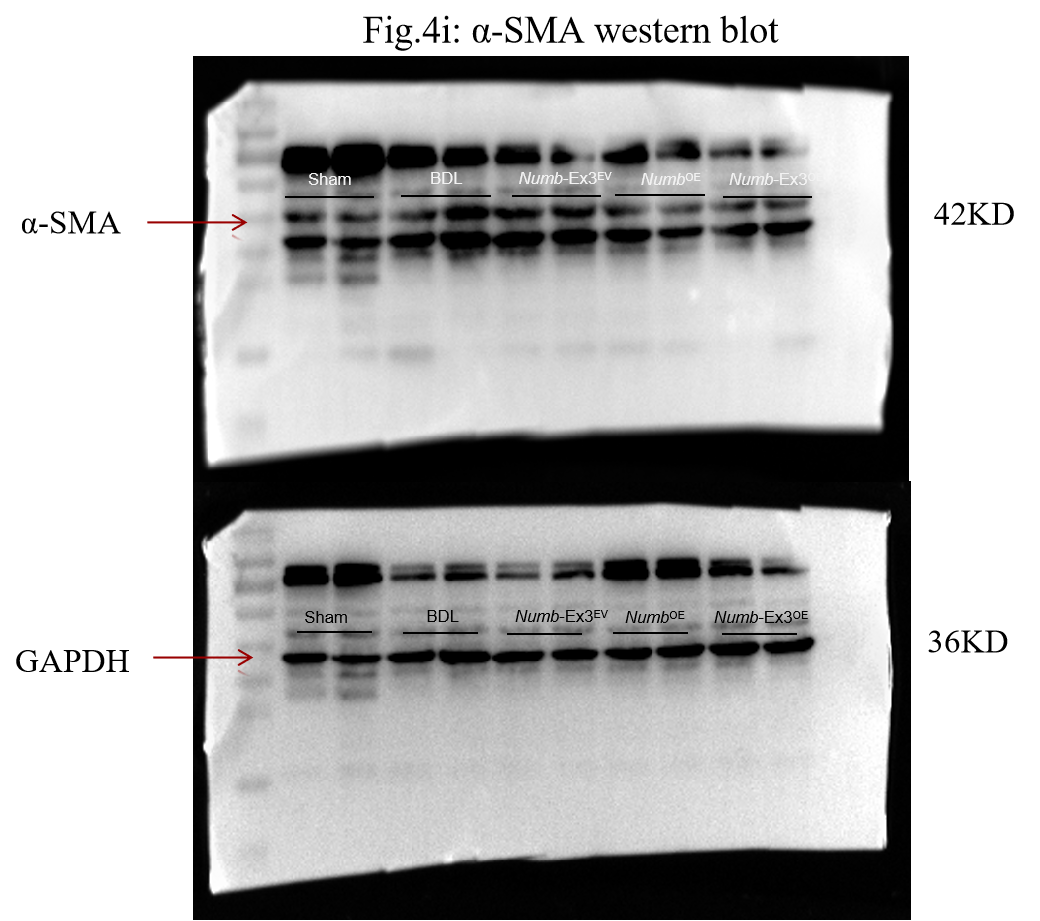


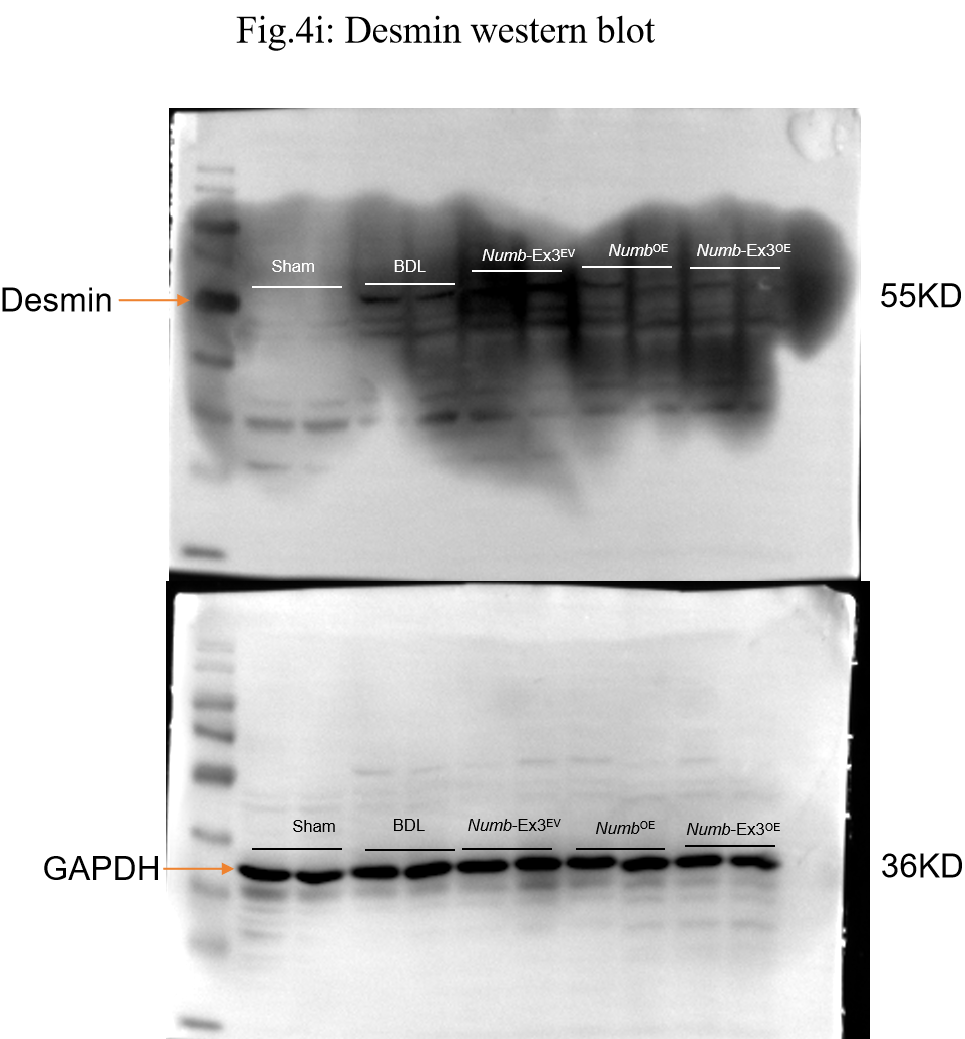

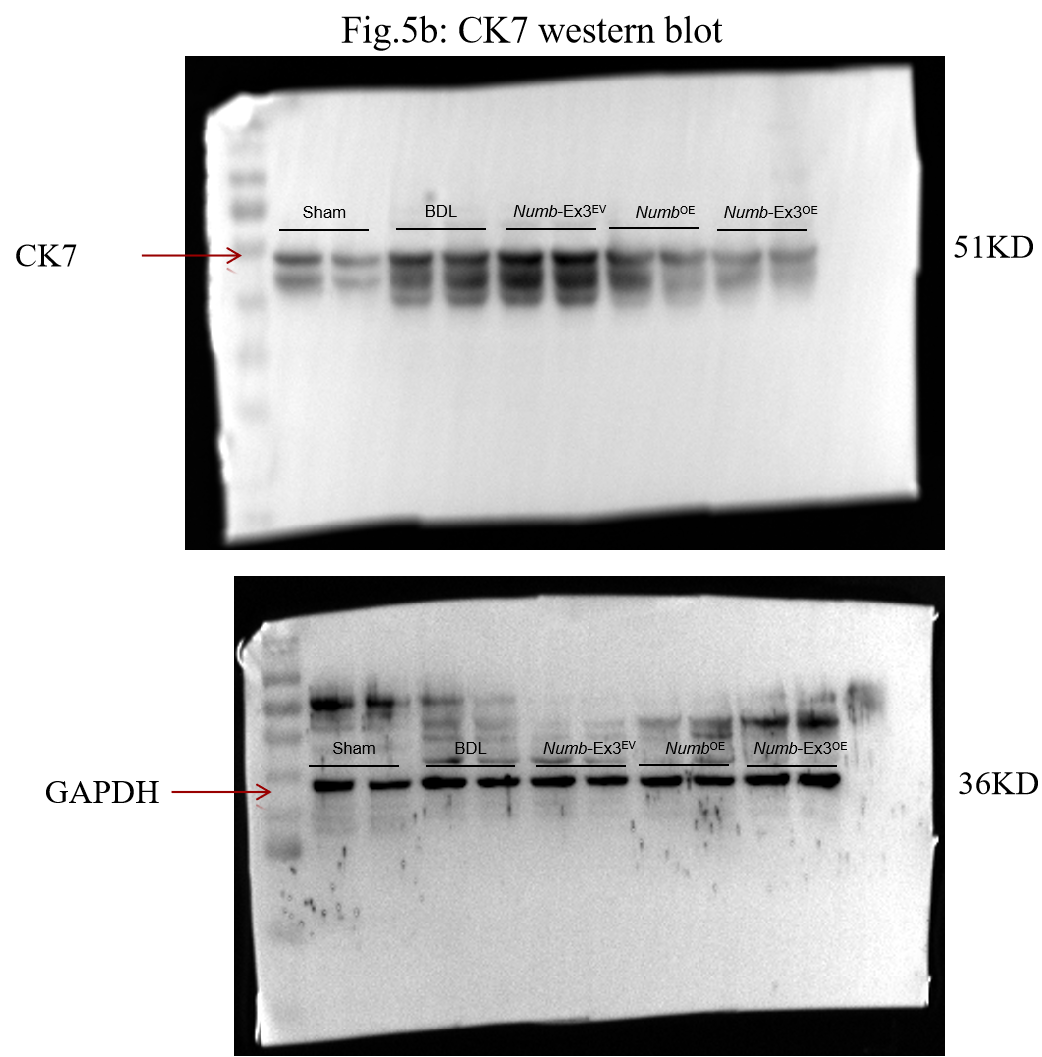


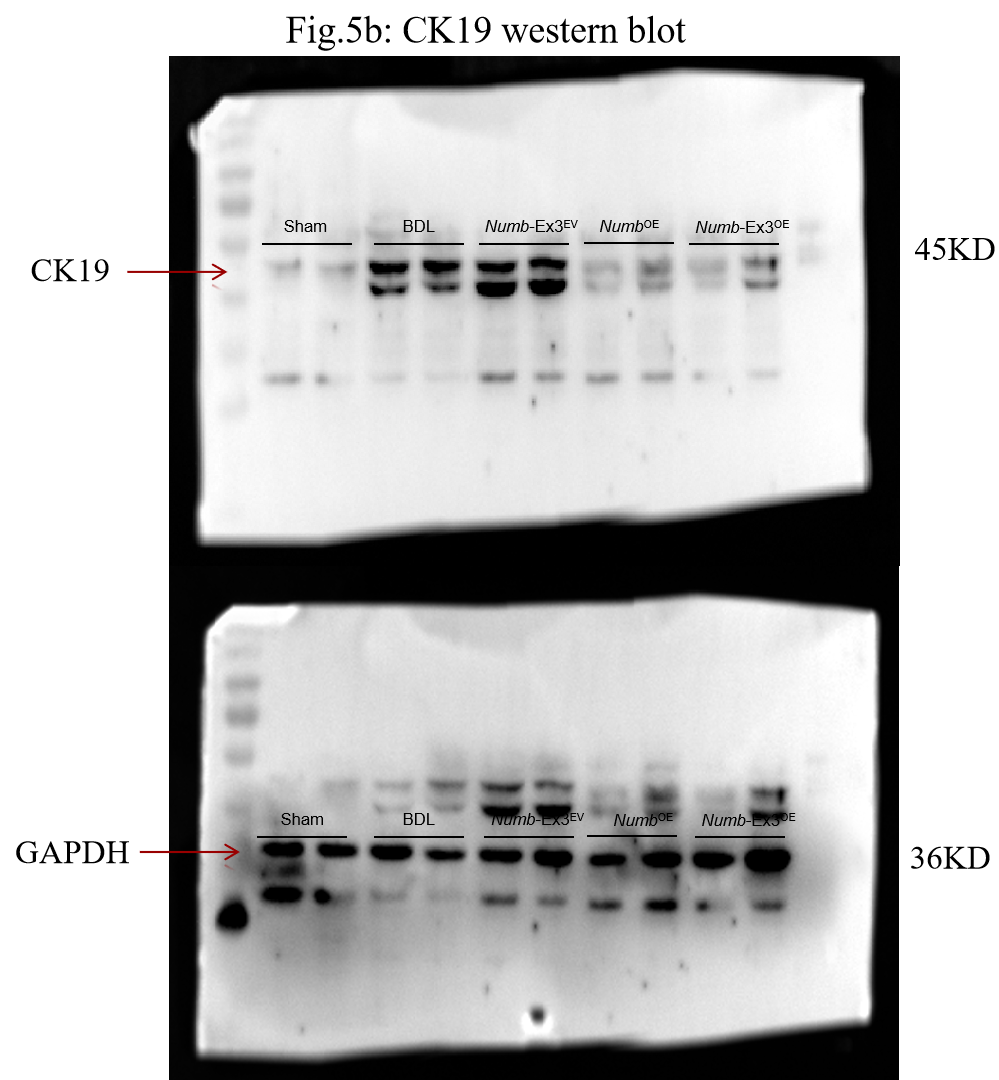


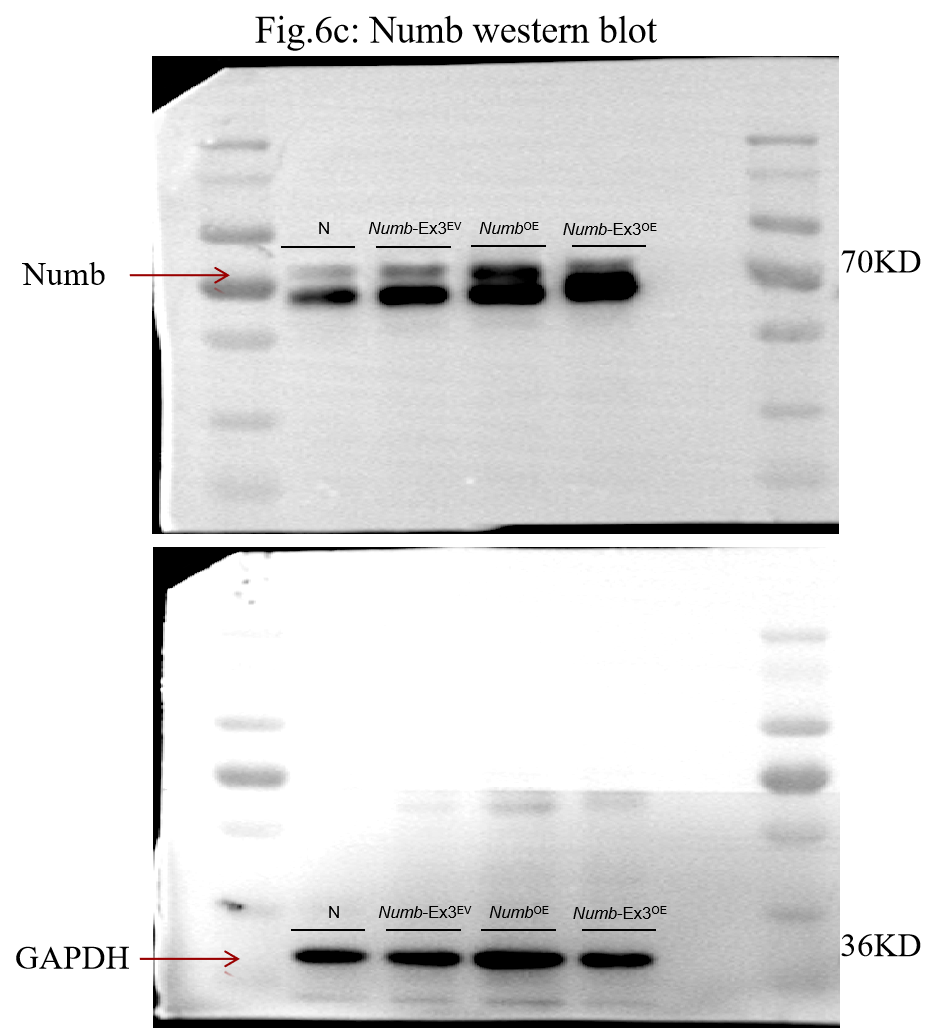


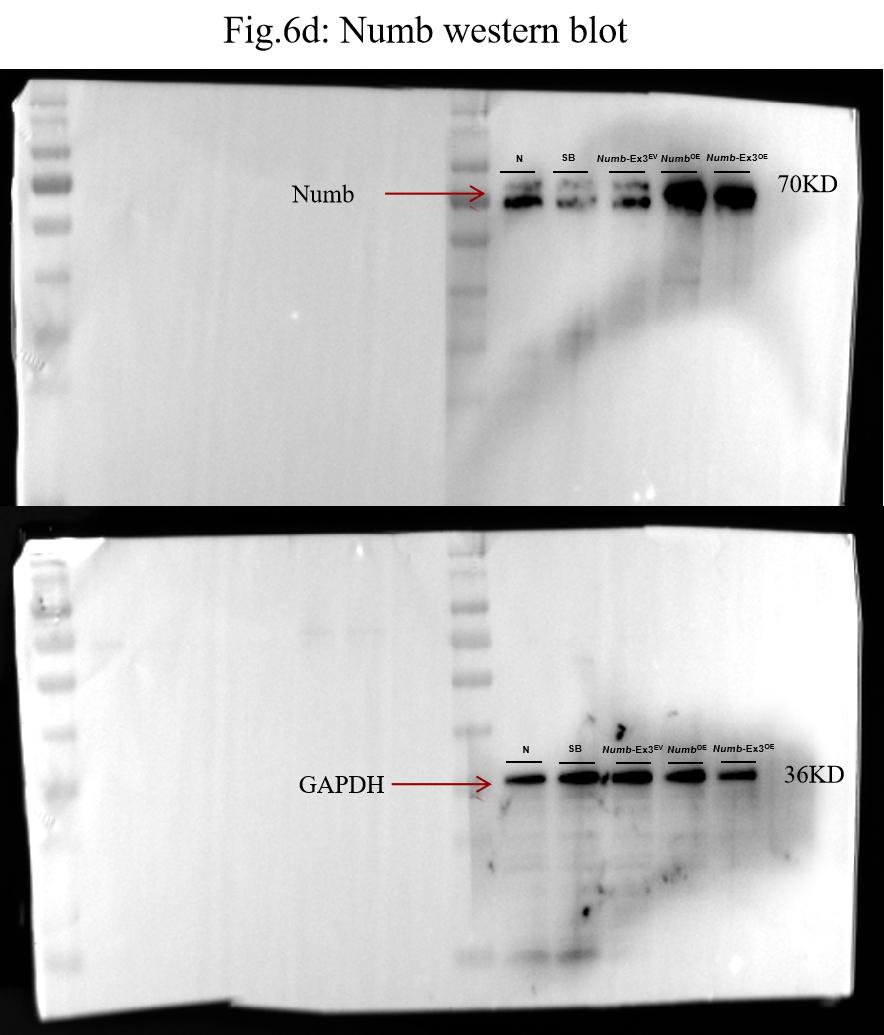


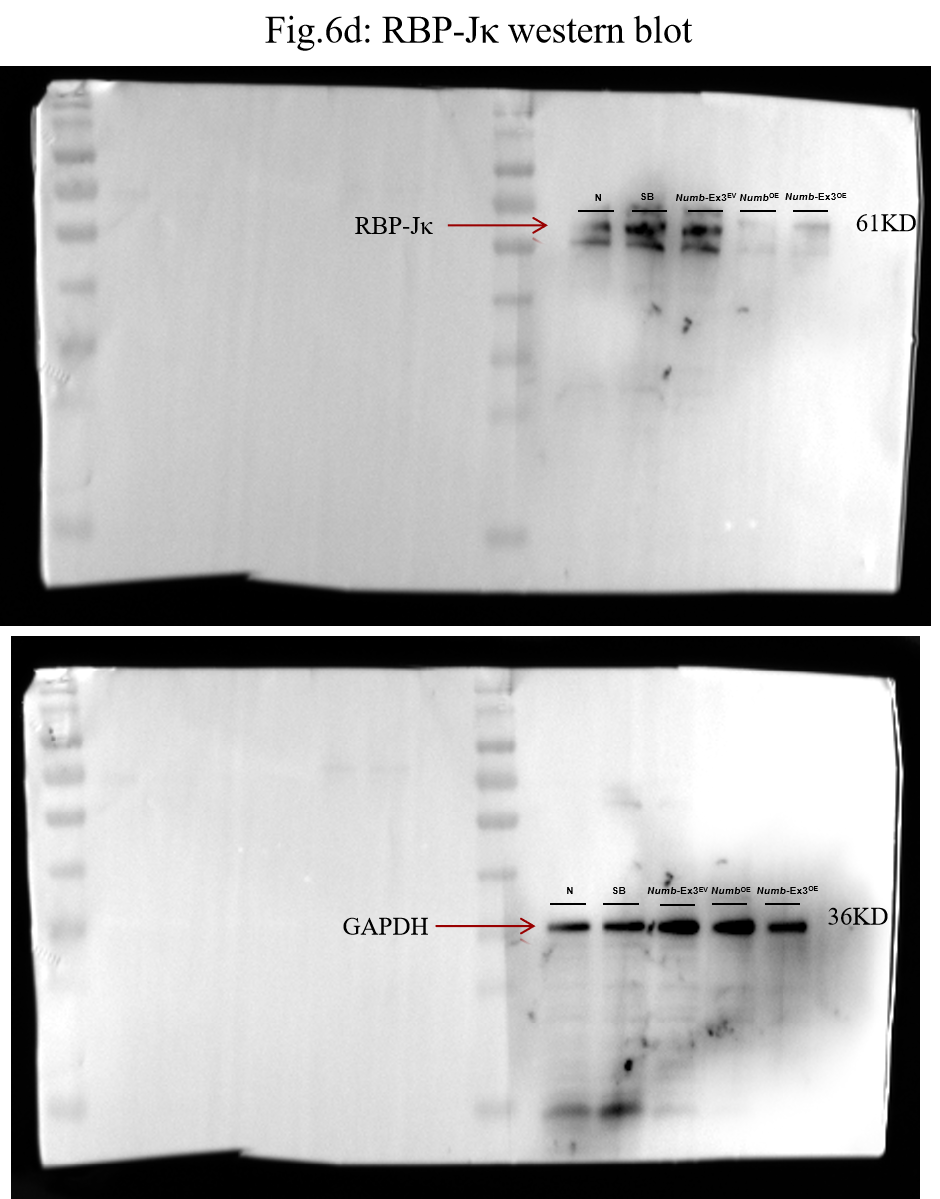


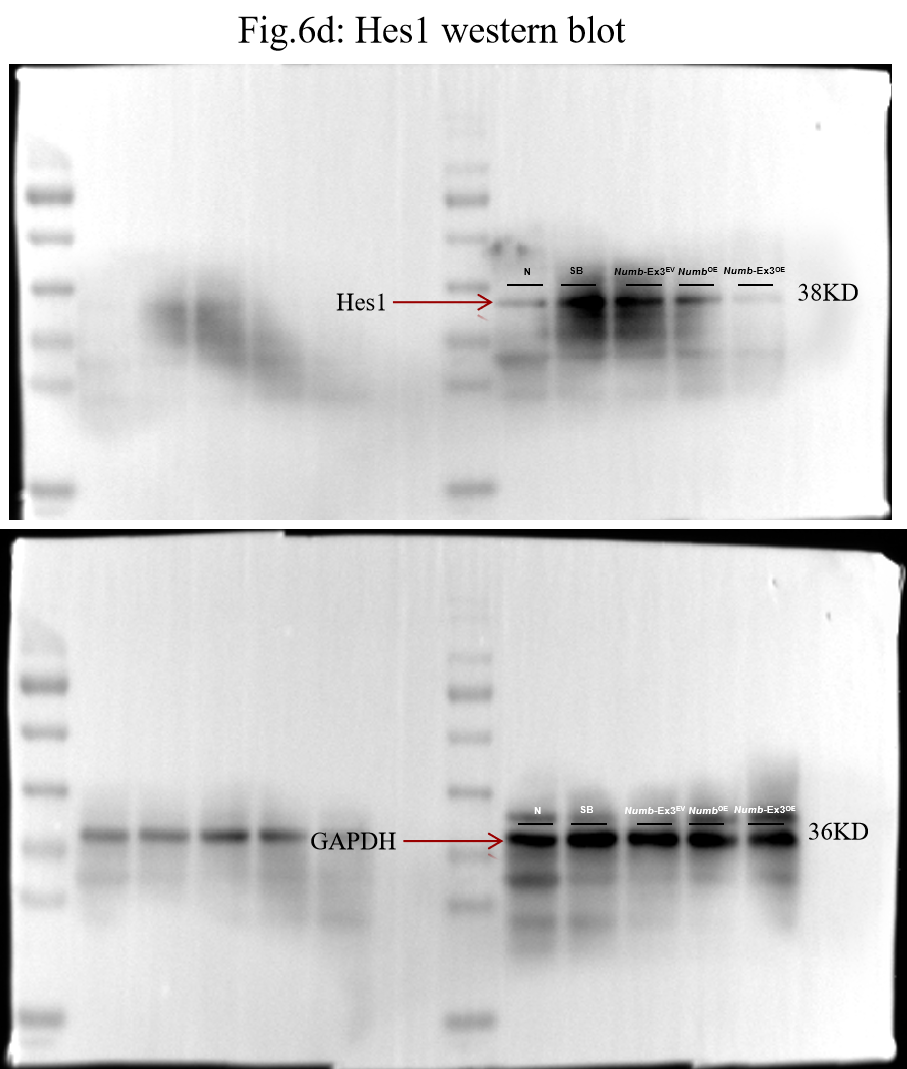


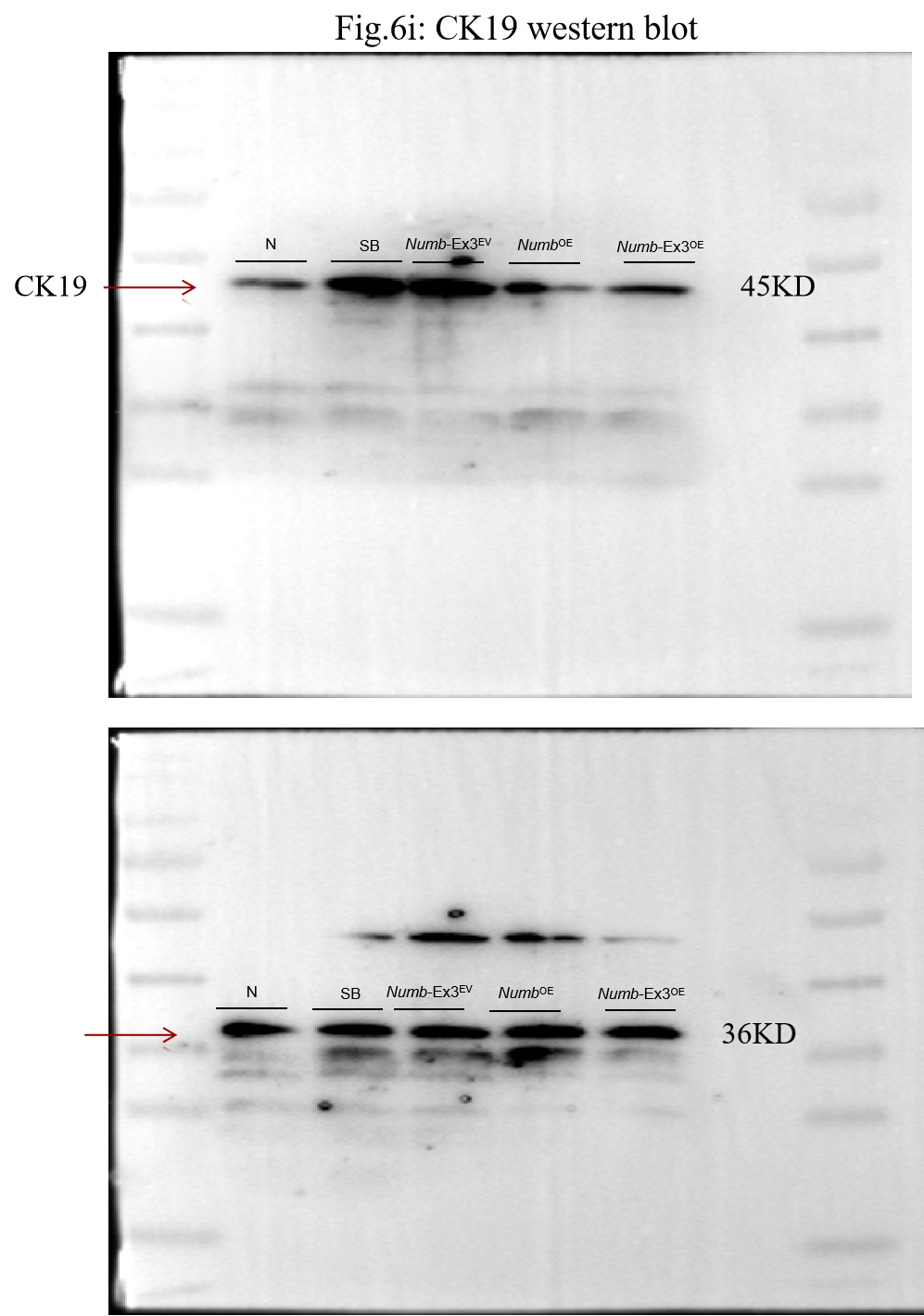


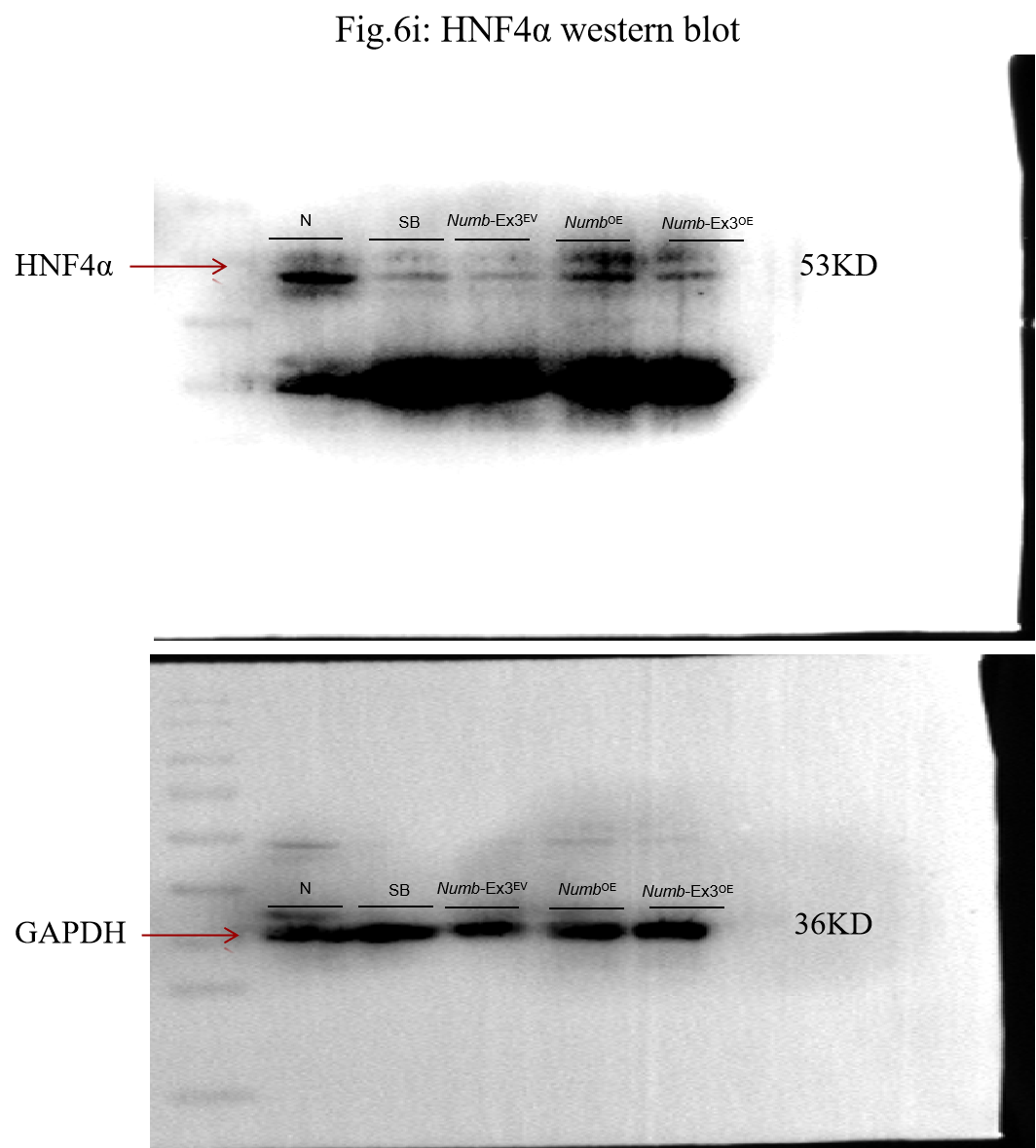


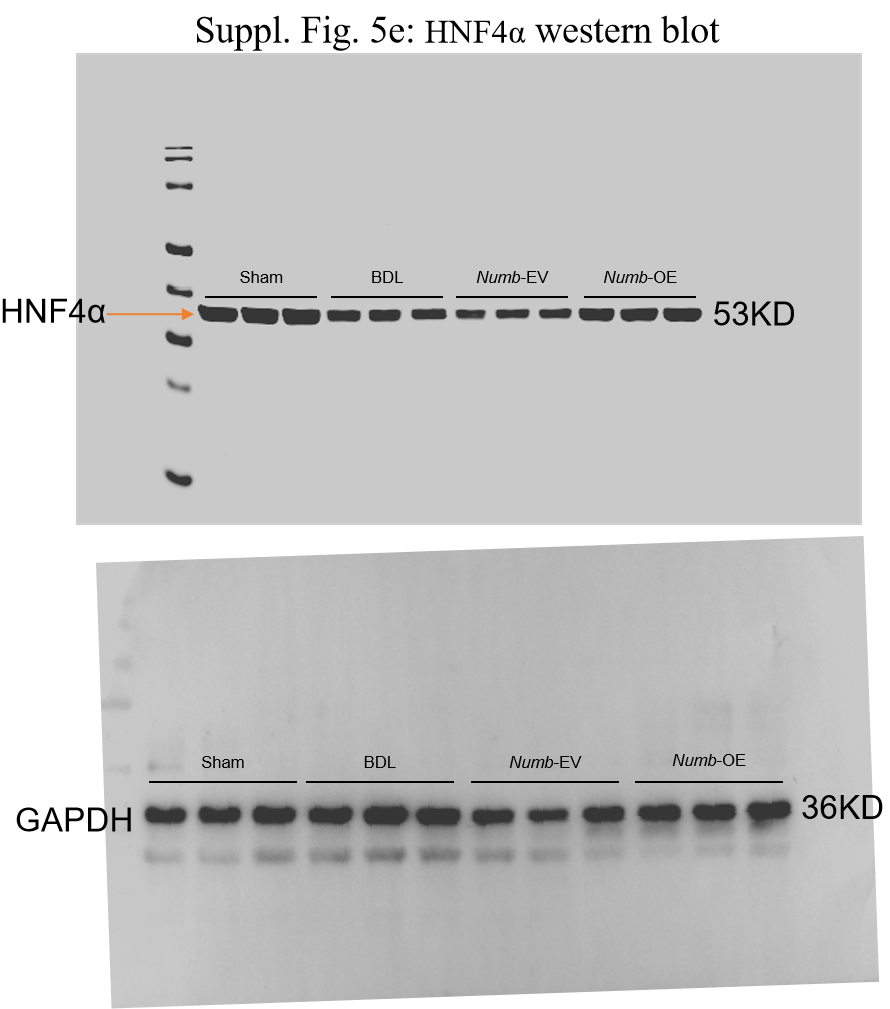

Supplement: Supplementary file 1 — Supplementary Material 1 [file 41598_2025_23696_MOESM1_ESM.docx]
